# Sox11 Is Required to Maintain Proper Levels of Hedgehog Signaling during Vertebrate Ocular Morphogenesis

**DOI:** 10.1371/journal.pgen.1004491

**Published:** 2014-07-10

**Authors:** Lakshmi Pillai-Kastoori, Wen Wen, Stephen G. Wilson, Erin Strachan, Adriana Lo-Castro, Marco Fichera, Sebastiano A. Musumeci, Ordan J. Lehmann, Ann C. Morris

**Affiliations:** 1 Department of Biology, University of Kentucky, Lexington, Kentucky, United States of America; 2 Departments of Ophthalmology and Medical Genetics, University of Alberta, Edmonton, Alberta, Canada; 3 Department of Neuroscience, Pediatric Neurology Unit, “Tor Vergata” University of Rome, Rome, Italy; 4 Laboratory of Medical Genetics, IRCCS Associazione Oasi Maria Santissima, Troina, Italy, and Medical Genetics, University of Catania, Catania, Italy; 5 Unit of Neurology, IRCCS Associazione Oasi Maria Santissima, Troina, Italy; Stanford University School of Medicine, United States of America

## Abstract

Ocular coloboma is a sight-threatening malformation caused by failure of the choroid fissure to close during morphogenesis of the eye, and is frequently associated with additional anomalies, including microphthalmia and cataracts. Although Hedgehog signaling is known to play a critical role in choroid fissure closure, genetic regulation of this pathway remains poorly understood. Here, we show that the transcription factor Sox11 is required to maintain specific levels of Hedgehog signaling during ocular development. Sox11-deficient zebrafish embryos displayed delayed and abnormal lens formation, coloboma, and a specific reduction in rod photoreceptors, all of which could be rescued by treatment with the Hedgehog pathway inhibitor cyclopamine. We further demonstrate that the elevated Hedgehog signaling in Sox11-deficient zebrafish was caused by a large increase in *shha* transcription; indeed, suppressing Shha expression rescued the ocular phenotypes of *sox11* morphants. Conversely, over-expression of *sox11* induced cyclopia, a phenotype consistent with reduced levels of Sonic hedgehog. We screened DNA samples from 79 patients with microphthalmia, anophthalmia, or coloboma (MAC) and identified two novel heterozygous *SOX11* variants in individuals with coloboma. In contrast to wild type human *SOX11* mRNA, mRNA containing either variant failed to rescue the lens and coloboma phenotypes of Sox11-deficient zebrafish, and both exhibited significantly reduced transactivation ability in a luciferase reporter assay. Moreover, decreased gene dosage from a segmental deletion encompassing the *SOX11* locus resulted in microphthalmia and related ocular phenotypes. Therefore, our study reveals a novel role for Sox11 in controlling Hedgehog signaling, and suggests that *SOX11* variants contribute to pediatric eye disorders.

## Author Summary

Ocular coloboma is a condition in which tissue is missing from a portion of the eye due to its abnormal development. Coloboma is also frequently associated with additional anomalies, including microphthalmia (abnormally small eye) and cataracts. Although some of the genes that cause coloboma have been identified, in the majority of cases the underlying genetic cause has not been determined. One pathway that has been implicated in coloboma is the Hedgehog (Hh) signaling pathway. In this study, we have taken advantage of the ability to titrate levels of gene expression in zebrafish to demonstrate for the first time that the transcription factor Sox11 is required to limit levels of Hedgehog (Hh) signaling during ocular development. We show that in the absence of Sox11, levels of the Sonic Hedgehog (Shh) ligand are greatly elevated, which disrupts the proper patterning of the optic stalk and optic vesicle, resulting in coloboma. We also provide evidence that *SOX11* dosage changes or mutations contribute to human coloboma, microphthalmia, and rod photoreceptor dysfunction. Thus, our work establishes a novel link between Sox11 and Hh signaling, and suggests that mutations in *SOX11* contribute to pediatric eye disorders such as coloboma.

## Introduction

Ocular coloboma arises when the embryonic choroid fissure in the ventral optic cup fails to close. It can cause significant pediatric visual impairment [1], and is often associated with other ocular abnormalities such as microphthalmia or anophthalmia (collectively referred to as MAC). Coloboma may also be observed in conjunction with dysgenesis of the anterior segment (front portion of the eye) or optic nerve, lenticular defects (such as cataract), or systemic congenital malformation syndromes [2]. In addition to phenotypic heterogeneity, coloboma is genetically heterogeneous, exhibiting differing patterns of inheritance, variable expressivity, and reduced penetrance [2].

Among the signaling pathways that converge to regulate ocular morphogenesis, Hedgehog (Hh) signaling has a critical role and acts reiteratively during eye development [3]. Hh signaling from the midline promotes the segregation of the single eye field into two optic primordia, and is required for the correct proximodistal and dorsoventral patterning of the optic vesicle [3]–[5]. Once the optic cup has formed, intraretinal Hh signaling regulates the differentiation of retinal progenitor cells [3]. Given its central role in eye development, it is unsurprising that mutations in genes encoding Hh pathway ligands (SHH) or targets (PAX2, VAX1) are associated with congenital ocular malformations in humans [6]–[9]. However, these mutations account for only a minority of patients; for the majority of MAC cases, the molecular defect has yet to be identified. Because of their potency, the spatiotemporal levels of Hh ligands must be tightly regulated throughout eye development; yet, very little is known about the factors that restrict their expression during oculogenesis. Such factors would represent excellent candidate genes for human coloboma and associated ocular defects, and potentially could be used to influence Hh signaling.

Here, we focus on the role of the SRY-box transcription factor Sox11 during eye development. Sox11 is a member of the group C family of SOX proteins, which also includes Sox4 and Sox12 [10]. Sox11 is required for a variety of processes, including organogenesis and neurogenesis, craniofacial and skeletal development [10], as well as being implicated in carcinogenesis (including mantle cell lymphoma, medulloblastoma, and glioblastoma) [10], [11]. Expression and functional studies support a role for Sox11 during several stages of eye development. In the mouse, Sox11 is expressed in the optic cup and periocular mesenchyme during early eye development, and in the developing lens and retina at later stages [12], [13]. In the zebrafish retina, we previously found that Sox11 is upregulated in rod progenitor cells during rod photoreceptor regeneration [14]. *Sox11^−/−^* mice exhibit ocular abnormalities such as anterior segment dysgenesis, microphthalmia, a persistent lens stalk, delayed lens formation, and coloboma [13]. Finally, some human chromosomal rearrangements resulting in ocular abnormalities have been mapped to the vicinity of the *SOX11* locus at chromosome 2p25.2 [15]–[18]. These data together suggested intriguing roles for Sox11 in ocular morphogenesis and rod photoreceptor differentiation, however the underlying mechanisms were undefined.

In this study, we inhibited Sox11 activity in zebrafish embryos, and based on the resultant phenotypes demonstrate that the function of Sox11 in regulating lens development and choroid fissure closure is evolutionarily conserved, and that Sox11 is required for rod photoreceptor differentiation. We demonstrate that elevated Hh signaling causes the ocular phenotypes in Sox11-deficient zebrafish, and that Sox11 is required to repress expression of the Sonic hedgehog gene (*shha*). Finally, we identify *SOX11* variants with reduced transactivation ability in MAC patients, and in parallel demonstrate that decreased *SOX11* gene dosage results in congenital ocular abnormalities. In revealing a previously uncharacterized role for Sox11 upstream of Hh signaling, these studies may substantially extend our understanding of additional Sox11-dependent developmental and pathologic processes.

## Results

### Expression of *sox11a/b* during ocular development

**Figure 1:**
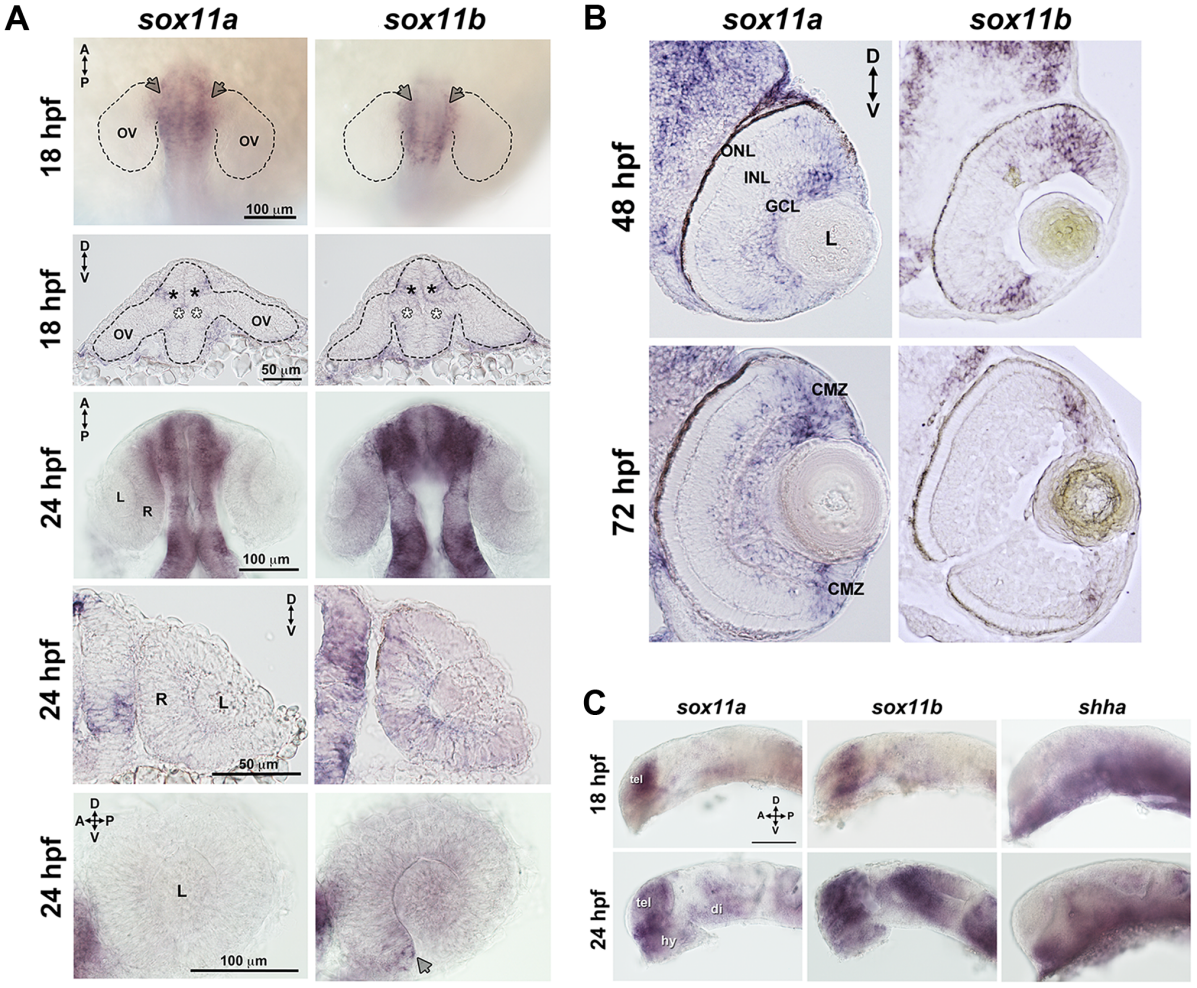
Developmental expression of *sox11*. In situ hybridization with antisense probes for *sox11a*, *sox11b*, and *shha* was performed on whole embryos or on tissue sections at the indicated time points. (A) *Sox11a* and *sox11b* were expressed in the diencephalon adjacent to the optic vesicle (arrows in top row and asterisks in second row) at 18 hpf (top two rows) and 24 hpf (third and fourth rows). *Sox11a* expression was not detected in the lens or retina at 24 hpf (bottom left). *Sox11b* was expressed in a patch of cells in the ventronasal retina (arrow, bottom right) and more diffusely across the rest of the retina and lens. Top and third rows are dorsal views of flat-mounted embryos. Second and fourth rows are frontal sections through the head. Bottom panels are lateral views of dissected eyes; (n = 20 embryos examined per time point, 3 independent repeats). (B) Transverse sections through the eye at 48 hpf (top) and 72 hpf (bottom). *Sox11a* expression was detected in the ganglion cell layer (GCL) and in few sporadic cells in the inner nuclear layer (INL); *sox11b* expression was observed in scattered cells across the central retina and in the peripheral retina. At 72 hpf, *sox11a* expression persisted in the GCL and in some cells in the INL; *sox11a* and *sox11b* were also expressed in the persistently neurogenic ciliary marginal zone (CMZ); n = 20 embryos examined per time point, 3 independent repeats. (C) Expression patterns of *sox11a* (left), *sox11b* (center), and *shha* (right) in the developing brain at 18 hpf (top) and 24 hpf (bottom). The eye was removed to better image the brain. Expression of *sox11a* and *sox11b*, but not *shha*, was observed in the telencephalon. Expression of all three genes was detected in the hypothalamus and ventral diencephalon at 24 hpf (n = 20 embryos examined per time point, 3 independent repeats). Scale bar = 100 µm; D, dorsal; V, ventral; A, anterior; P, posterior; hpf, hours post fertilization; OV, optic vesicle; L, lens; R, retina; GCL, ganglion cell layer; INL, inner nuclear layer; ONL, outer nuclear layer; CMZ, ciliary marginal zone; tel, telencephalon; hy, hypothalamus; di, diencephalon.

Zebrafish possess two orthologs of mammalian Sox11, which are expressed in overlapping and distinct domains ([19]–[21], this study). Previous studies have shown that both *sox11a* and *sox11b* are maternally expressed prior to the midblastula transition, and are expressed in the region of the anterior neural plate that gives rise to the diencephalon at the onset of the segmentation period [20]. Using in situ hybridization with paralog-specific probes, we investigated the expression of *sox11a* and *sox11b* both within the forebrain during optic cup formation, and in the eye at later stages of retinal development. At 18 hours post fertilization (hpf), we detected expression of *sox11a* and *sox11b* in the telencephalon, and in the dorsal “corner” formed by the diencephalon and the evaginated optic stalk/optic vesicle (top panel arrows and second row closed asterisks, Figure 1A). We also detected faint expression of *sox11a* and *sox11b* at the ventral hinge of the optic stalk/optic vesicle axis (open asterisks, Figure 1A). However, we did not detect expression of *sox11a* or *sox11b* within the optic vesicle itself (Figure 1A). At 24 hpf, *sox11a/b* expression persisted in the diencephalon adjacent to the retina and in the telencephalon, and both paralogs were also expressed in the hypothalamus (Figures 1A, C). Within the developing retina at 24 hpf, *sox11b* was expressed diffusely across the lens and retinal neuroepithelium, and was distinctly visible in a small cluster of cells in the ventro-nasal retina (arrow, bottom right panel, Figure 1A), corresponding to the location at which retinal neurogenesis initiates [22]. As retinal development proceeded, *sox11a* expression was observed in the ganglion cell layer (GCL) at 48 hpf, whereas the expression of *sox11b* was detected in a few scattered cells across the central retina but was mostly restricted to the undifferentiated peripheral retina (Figure 1B; [14]). By 72 hpf, when retinal neurogenesis was mostly complete, both *sox11a* and *sox11b* were predominantly expressed in the persistently neurogenic ciliary marginal zone (Figure 1B; [14]); expression of *sox11a* also persisted in the GCL and in some cells in the inner half of the inner nuclear later (INL). Interestingly, the expression domains of both *sox11* paralogs were adjacent to regions of *shha* expression in the ventral diencephalon at 18 and 24 hpf (Figure 1C), whereas at 48 hpf *sox11a* expression overlapped with the previously described location of *shha* in the GCL [23], [24].

### Knockdown of *sox11a/b* causes abnormal ocular morphogenesis

**Figure 2:**
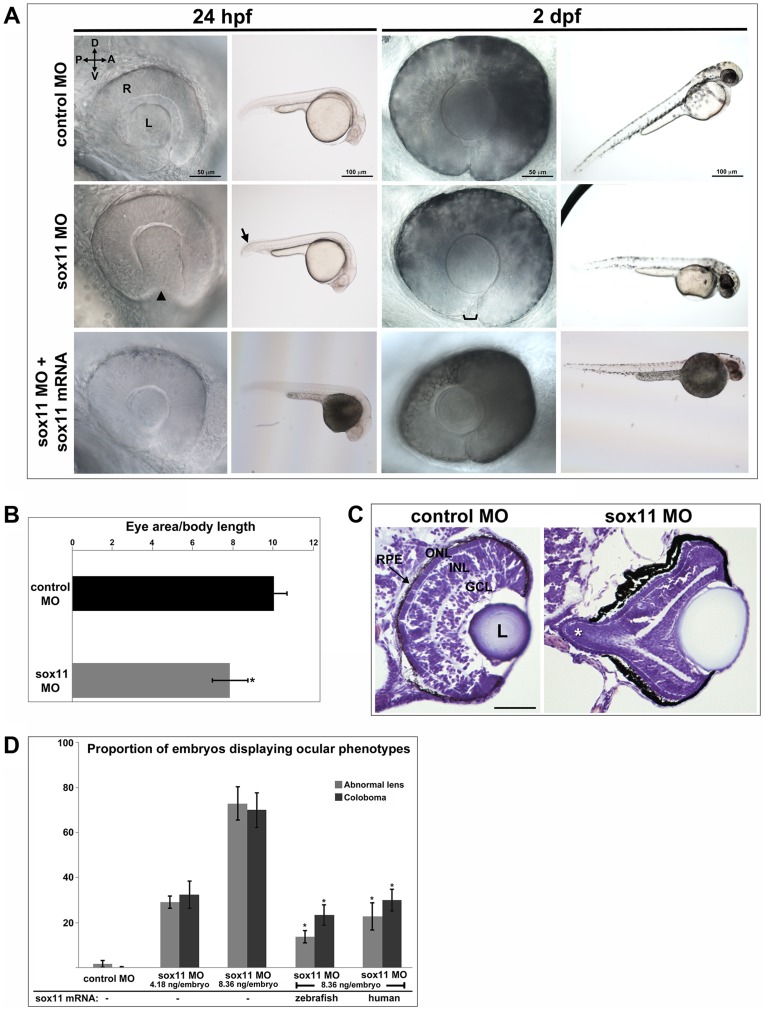
Sox11 knockdown disrupts ocular morphogenesis and causes coloboma in zebrafish. (A) Representative eye and body images of control and *sox11* morphants (taken from the set of embryos analyzed in (D). At 24 hpf, approximately 70% of *sox11* morphants displayed a malformed lens (arrowhead) and a posterior kink in the tail (arrow). At 2 dpf, a similar proportion of *sox11* morphants displayed coloboma (bracket), and had a hypopigmented and underdeveloped ventral retina. Both the abnormal lens and coloboma phenotypes were rescued with co-injection of wild type zebrafish *sox11* mRNA (bottom row). (B) *Sox11* morphants were microphthalmic at 24 hpf. Eye area was normalized to body length (*p<0.0001, Student's *t*-test; control MO: n = 10 embryos examined; *sox11* MO: n = 12 embryos examined, 3 independent repeats). (C) Sections of 72 hpf control (left) and *sox11* morphant eyes (right) stained with cresyl violet revealed the extrusion of the retina into the brain through the open choroid fissure of *sox11* morphants (asterisk); n = 6 individuals examined per group. The thickened appearance of the dorsal RPE in the *sox11* morphant retina is a staining artifact and was not observed in fresh tissue sections. Scale bar = 50 µm. (D) Injection of zebrafish and human *sox11* mRNA rescued the ocular phenotypes in *sox11* morphants. Number of embryos analyzed: 24 hpf control MO, 4.18 ng/embryo, n = 1007; 2 dpf control MO, 4.18 ng/embryo, n = 1001; 24 hpf *sox11* MO, 4.18 ng/embryo, n = 309; 2 dpf *sox11* MO, 4.18 ng/embryo, n = 294; 24 hpf *sox11* MO, 8.36 ng/embryo, n = 559; 2 dpf *sox11* MO, 8.36 ng/embryo, n = 392; 24 hpf *sox11* MO 8.36 ng/embryo plus 2.0 ng/embryo zebrafish *sox11* mRNA, n = 185; 2 dpf *sox11* MO, 8.36 ng/embryo plus 2.0 ng/embryo zebrafish *sox11* mRNA, n = 167; 24 hpf *sox11* MO, 8.36 ng/embryo plus 0.3 ng/embryo human *SOX11* mRNA, n = 130; 2 dpf *sox11* MO, 8.36 ng/embryo plus 0.3 ng/embryo human *SOX11* mRNA, n = 125. Three biological replicates were performed for all experiments. (*p<0.001, Student's *t*- test). D, dorsal; V, ventral; A, anterior; P, posterior; L, lens; R, retina; hpf, hours post fertilization; dpf, days post fertilization; MO, morpholino; GCL, ganglion cell layer; INL, inner nuclear layer; ONL, outer nuclear layer; RPE, retinal pigmented epithelium.

To investigate the function of Sox11 paralogs during eye development, translation of *sox11a* and *sox11b* was blocked with morpholino oligonucleotides (MOs), whose efficiency and specificity were confirmed using a second *sox11* MO and a GFP reporter assay, respectively (Figures S1A, C). Zebrafish embryos were injected with *sox11a* and *sox11b* MOs simultaneously (hereafter referred to as *sox11* morphants), as co-inhibition of both paralogs induced the highest prevalence of ocular phenotypes (Figure S1B). At 24 hpf, 72.9±7.4% of *sox11* morphants displayed a misshapen, rudimentary, or absent lens (Figures 2A, S1B). *Sox11* morphant lenses mostly recovered to a spherical shape by 2 days post fertilization (dpf), however at this stage a similar proportion of morphants (70.0±7.7%) displayed coloboma (Figures 2A, S1B). *Sox11* morphant eyes were also hypopigmented ventrally, and microphthalmic compared to controls (Figure 2A, B). Histological sections revealed that the colobomatous retinas in *sox11* morphants frequently extruded through the open choroid fissure into the brain (Figure 2C). Approximately 54% (15 of 28 individuals examined) of *sox11* morphant retinas with coloboma also exhibited poor or reduced retinal lamination, suggesting a delay in retinal differentiation. In contrast, of the *sox11* morphant retinas that did not display coloboma, only 14% were poorly laminated (4 of 29). This suggests that similar mechanisms may underlie the ocular morphogenesis and retinal developmental defects observed in *sox11* morphants with coloboma. The presence of the coloboma prevented the retinal pigmented epithelium (RPE) from completely enclosing the posterior eye (Figure 2C), which likely accounts for the hypopigmented appearance of the ventral portion of the eye when viewed laterally (Figure 2A). This coloboma phenotype was reminiscent of the zebrafish *blowout* mutant, which has a mutation in *patched2* (formerly named *patched1*), a negative regulator of Hh signaling [25], [26]. In addition to the ocular phenotypes, *sox11* morphants also frequently displayed a downward kink of the tail, as well as brain abnormalities such as widened ventricles, likely reflecting Sox11's expression and function in the posterior somites and developing brain, respectively [10], [20]. All of the morphant phenotypes were rescued by injection of wild type *sox11a* and *sox11b* mRNA, consistent with the morpholinos being specific for Sox11 (Figure 2A, D). Importantly, these phenotypes were also rescued by injection of human *SOX11* mRNA, indicating that the function of Sox11 in regulating early eye development is evolutionarily conserved (Figure 2D).

One mechanism that has been suggested to contribute to optic fissure closure defects is overproliferation of progenitor cells within the presumptive neural retina [27]. To determine whether changes in mitotic activity underlie the lens and coloboma phenotypes in *sox11* morphants, we immunolabeled retinal sections from control and *sox11* morphants with an antibody to phosphohistone H3 (PH3). We observed a modest but significant increase in the number of PH3-positive cells in *sox11* morphant optic vesicle and retinas at 18 and 24 hpf, and a larger increase in proliferation relative to controls at 48 and 72 hpf (Figure S2C, D). However, the excess PH3-positive cells were not clustered in the ventral retina, optic stalk, or lens at 24 hpf (Figure S2D), by which time the abnormal ocular phenotypes are already apparent. Therefore, we conclude that overproliferation likely does not underlie the early ocular phenotypes of *sox11* morphants. We also performed TUNEL staining on sections from control and *sox11* morphant retinas (Figure S2A, B). This analysis revealed a variable but significant increase in TUNEL-positive cells in the optic vesicle of *sox11* morphants at 18 hpf. At 24 hpf, we did not detect elevated apoptosis in the retina or optic stalk of Sox11-deficient embryos. However, we did observe a significant increase in TUNEL-positive cells in the anterior lens of *sox11* morphants, which persisted through 72 hpf (Figure S2A, B). This increase in apoptotic cells in the lens may be related to the abnormal lens morphology we observed by light microscopy (Figure 2A). Finally, we observed an increase in TUNEL-positive cells in the colobomatous tissue of *sox11* morphant retinas at 48 hpf (Figure S2B), indicating that this abnormal ocular structure negatively impacted the survival of the cells within it.

### *Sox11* morphants possess fewer mature rod photoreceptors

**Figure 3:**
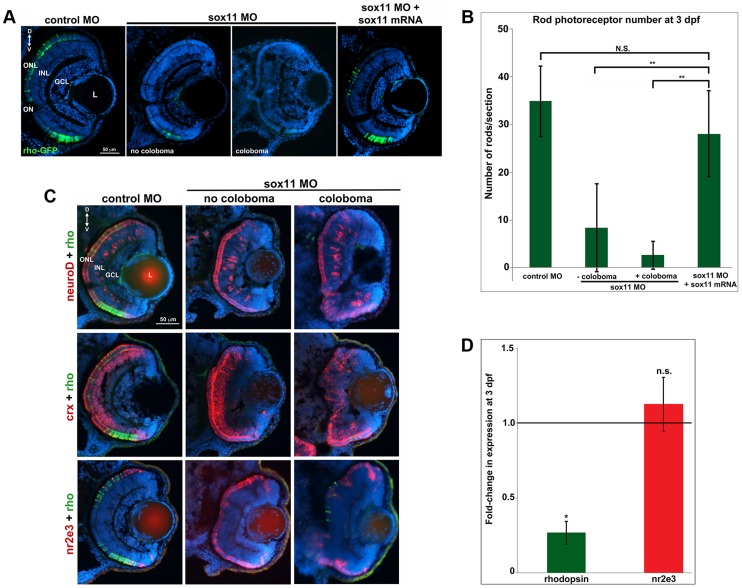
*Sox11* morphants lack mature rod photoreceptors. (A) Representative transverse retinal sections from XOPS-GFP zebrafish injected with control, *sox11* MO, or *sox11* MO plus zebrafish *sox11* mRNA at 3 dpf (from the set of individuals analyzed in (B). Even *sox11* morphants with well-laminated retinas and no evidence of coloboma (second panel) displayed greatly reduced numbers of mature rods compared to controls (left panel). Co-injection of wild type zebrafish *sox11* mRNA (right panel) rescued the rod deficiency at 3 dpf. (B) Quantification of the number of rod photoreceptors/section. Number of embryos analyzed: control MO, n = 25; *sox11* MO without coloboma, n = 25; *sox11* MO with coloboma, n = 25; *sox11* MO plus zebrafish *sox11* mRNA, n = 17 (**p<0.00001; n.s., p>0.05, Student's *t*- test). (C) Two-color fluorescent in situ hybridization (FISH) for *neuroD*, *crx*, *nr2e3*, and *rhodopsin* expression in control and *sox11* morphants with and without coloboma at 3 dpf. Expression of the rod lineage genes *neuroD* (top), *crx* (middle), and *nr2e3* (bottom) was qualitatively normal in *sox11* morphants with or without coloboma. However, *rhodopsin* expression (green) was greatly reduced compared to control morphants (left column). Number of embryos analyzed: n = 14 per group, 3 independent biological replicates. (D) Quantitative RT-PCR (qPCR) performed on mRNA from control and *sox11* morphant heads at 3 dpf revealed a significant decrease in *rhodopsin* expression in *sox11* morphants compared to controls. However, *nr2e3* transcript levels were not significantly different between control and *sox11* morphants. Relative transcript abundance was normalized to *atp5h* levels and is presented as the mean fold-change in expression relative to controls (n = 30 embryos per group, 3 independent biological replicates). *p<0.003; n.s, p>0.05, Student's *t*-test. D, dorsal; V, ventral; L, lens; dpf, days post fertilization; MO, morpholino; GCL, ganglion cell layer; INL, inner nuclear layer; ONL, outer nuclear layer; ON, optic nerve.

Given that expression of *sox11a/b* is upregulated in adult zebrafish rod progenitor cells during rod photoreceptor regeneration [14], we investigated whether Sox11-deficient embryos displayed altered rod development. Since we found that a significant proportion of *sox11* morphant retinas with coloboma also displayed poor lamination, indicating a potential delay in retinal development, for analysis we divided the *sox11* morphants into those with and without coloboma. This approach minimized the potential secondary effects on retinal development from the ocular morphogenetic defect masking any additional role for Sox11 in retinal neurogenesis. Using immunohistochemistry with cell-type specific antibodies, we found that *sox11* morphants without coloboma (approximately 30% of morphant embryos) possessed well-laminated retinas with normal numbers of ganglion, amacrine, horizontal, and bipolar cells, Müller glia, and cone photoreceptors at 72 hpf (Figure S3A, B). In contrast, when control and *sox11* MOs were injected into a rod photoreceptor-GFP transgenic reporter line [28], we observed a significant reduction in mature rod photoreceptors in *sox11* morphant retinas without coloboma at 3 dpf (control embryos, 34.9±7.4rods/section; *sox11* morphants, 8.7±8.9 rods/section; p<0.00001; Figure 3A, B). Furthermore, several retinal sections from *sox11* morphants contained no detectable GFP-positive rods at 3 dpf. The reduction in mature rod photoreceptors in *sox11* morphant retinas was confirmed by immunolabeling with the rod-specific antibody 4C12 (not shown), by fluorescent in situ hybridization (FISH) of retinal sections with a probe for *rhodopsin* (*rho*), and by quantitative RT-PCR (qPCR) for the *rho* transcript at 3 dpf (Figure 3C, D). Rod photoreceptor number could be rescued by injection of wild type *sox11* mRNA (Figure 3B), demonstrating that the reduction in rods was due to Sox11 deficiency. To determine whether depletion of Sox11 blocks specification of the rod photoreceptor fate, we conducted FISH on 3 dpf retinal sections from control and *sox11* morphants using probes for three genes associated with the rod photoreceptor lineage: *neuroD*, *crx*, and *nr2e3* [29]–[31]. Interestingly, we found that expression of all three rod lineage genes was qualitatively normal in *sox11* morphant retinas, even those with coloboma and poor lamination (Figure 3C). We also verified by qPCR that *nr2e3* transcript levels were not significantly different in *sox11* morphants and controls (Figure 3D) Therefore, these data suggest that Sox11 is required for the terminal differentiation, but not the specification, of rod photoreceptor cells. Because the window of rod photoreceptor differentiation is longer than that of cones or other retinal neurons [32], [33] we investigated whether rod photoreceptor number remained reduced in *sox11* morphants later in development. The number of rods in *sox11* morphant retinas was higher at 4 dpf than at 3 dpf, but remained significantly reduced relative to controls (*sox11* morphants, 15.9±2.9 rods/section; controls, 57.9±5.4 rods/section; p<0.001; Figure S3C). Taken together, these data suggest that terminal differentiation of rods requires Sox11.

### Sox11 negatively regulates Hedgehog signaling

**Figure 4:**
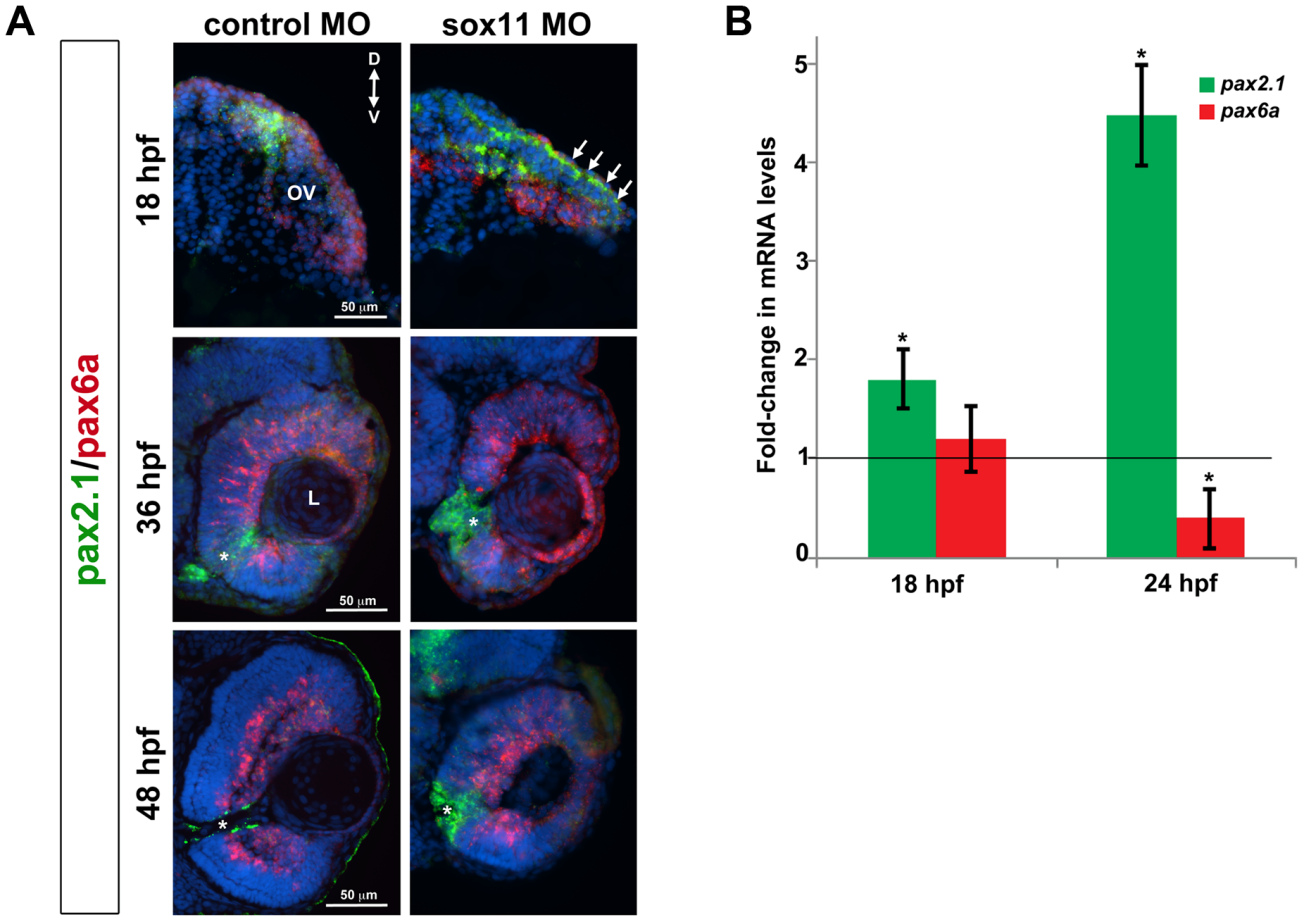
*Pax2.1* and *pax6a* expression is altered in *sox11* morphants. (A) Fluorescent in situ hybridization on transverse sections from control and *sox11* morphants with probes for *pax2.1* and *pax6a*. The expression domain of f *pax2.1* was expanded into the optic vesicle of *sox11* morphants at 18 hpf (top right, arrows), and there was a modest retraction of *pax6a* expression compared to controls (top left; number of embryos analyzed: control MO, n = 14; *sox11* MO, n = 13). At 36 and 48 hpf, in control retinas *pax2.1* expression decreased and was only observed lining the optic nerve (asterisk; left middle and bottom rows); in contrast, *pax2.1* expression was expanded and persisted around the open choroid fissure in *sox11* morphant retinas (asterisks, right middle and bottom rows). *Pax6a* expression in the retina of *sox11* morphants at 36 and 48 hpf appeared comparable to the control morphant retinas at this stage (number of embryos analyzed: 36 hpf control MO, n = 7; 36 hpf *sox11* MO, n = 12; 48 hpf control MO, n = 8; 48 hpf *sox11* MO, n = 14). (B) QPCR performed on mRNA from control and *sox11* morphant heads at 18 and 24 hpf revealed a significant increase in *pax2.1* expression at both 18 and 24 hpf, and a downregulation of *pax6a* expression at 24 hpf, in *sox11* morphants compared to controls. Relative transcript abundance was normalized to *atp5h* (18 hpf) or *gapdh* (24 hpf) levels and is presented as the mean fold-change in expression relative to controls (n = 50 embryos per group, 3 independent biological replicates. *p<0.05. D, dorsal; V, ventral; OV, optic vesicle; L, lens; hpf, hours post fertilization; MO, morpholino.

**Figure 5:**
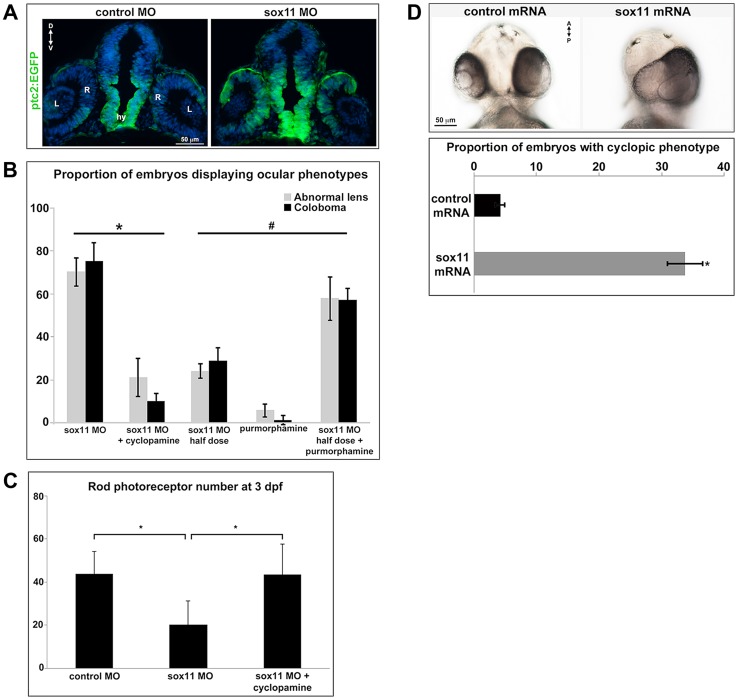
Sox11 negatively regulates Hedgehog (Hh) signaling. (A) Transverse retinal sections from 24 hpf *ptc2:EGFP* zebrafish embryos injected with control or *sox11* MO. *Sox11* morphants displayed elevated GFP expression in the brain as well as in the central and dorsal retina, and the dorsal RPE (number of embryos analyzed: control MO, n = 8; *sox11* MO, n = 10). (B) Treatment with the Hh inhibitor cyclopamine rescued the ocular phenotypes in *sox11* morphants. In contrast, treatment with the Hh agonist purmorphamine increased the prevalence of ocular phenotypes in embryos injected with a half dose of *sox11* MO. Number of embryos analyzed: 24 hpf *sox11MO* (plus 100% ethanol), n = 393; 2 dpf *sox11* MO (plus 100% ethanol), n = 319; 24 hpf *sox11* MO plus cyclopamine, n = 276; 2 dpf *sox11* MO plus cyclopamine, n = 263; 24 hpf half dose *sox11* MO (plus DMSO), n = 258; 2 dpf *sox11* MO half dose (plus DMSO), n = 241; 24 hpf uninjected plus purmorphamine, n = 83; 2 dpf uninjected plus purmorphamine, n = 81; 24 hpf half dose *sox11* MO plus purmorphamine, n = 291; 2 dpf half dose *sox11* MO plus purmorphamine, n = 270; 3 independent biological replicates. * and # p<0.0001, Fisher's exact test. (C) Treatment with cyclopamine rescued rod photoreceptor number in *sox11* morphants. Rods were counted in retinal cryosections from 3 dpf embryos. Number of embryos analyzed: control MO, n = 17; *sox11* MO, n = 20; *sox11* MO plus cyclopamine, n = 18; 3 independent replicates. *p = 0.02, Student's *t*-test). (D) Overexpression of zebrafish *sox11* increased the proportion of embryos with a cyclopic phenotype (right) compared to embryos injected with equimolar amounts of control *td*-*tomato* mRNA (left). Number of embryos analyzed: control mRNA, n = 168; *sox11* mRNA, n = 202, 3 independent biological replicates.*p<0.001, Fisher's exact test. D, dorsal; V, ventral; A, anterior; P, posterior; hpf, hours post fertilization; dpf, days post fertilization R, retina; hy, hypothalamus; L, lens; MO, morpholino.

As mentioned above, the coloboma phenotype of *sox11* morphants is similar to the zebrafish *blowout* mutant, in which increased Hedgehog signaling results in altered proximodistal patterning of the optic vesicle [25]. To determine whether a similar defect was present in *sox11* morphants, we performed FISH on retinal sections to examine the expression of *pax2a* and *pax6a*, which mark optic stalk and retinal territories, respectively. This revealed expansion of the *pax2a* domain in approximately 50% of *sox11* morphant embryos at 18 and 36 hpf, while at later stages (48 hpf), expression persisted around the open choroid fissure, whereas it was barely detectable in controls (Figure 4A). These expression changes were verified by qPCR at 18 and 24 hpf. Although we did not observe a concomitant decrease in *pax6a* expression in the optic vesicle at 18 hpf, there was a significant reduction in transcript levels detected by qPCR at 24 hpf (Figure 4B). To further test whether Hh signaling was elevated in *sox11* morphants, we made use of a recently described Hh signaling reporter line of zebrafish, which expresses GFP under the control of the *patched2* (*ptc2*) promoter [34]. Sections through the head of 24 hpf control and *sox11* morphants on the *ptc2:GFP* background revealed both an increase in GFP expression and an expansion of the GFP-positive domains in the brain, retina, and RPE of *sox11* morphants (Figure 5A). Taken together, these data strongly suggest that Hh signaling is indeed elevated in *sox11* morphants.

To directly test this hypothesis, control and *sox11* morphant embryos were treated from 5.5–13 hpf with the Hh inhibitor cyclopamine. This treatment window was chosen because it resulted in maximal rescue of colobomas in the *blowout* mutant [25]. The proportion of embryos displaying a malformed lens at 24 hpf (21.3±8.8%) or coloboma at 2 dpf (10.1±3.8%) was significantly reduced after cyclopamine treatment, compared to vehicle-treated *sox11* morphants (>70% for both phenotypes; p<0.0001; Figures 5B and S4A). Moreover, cyclopamine treatment significantly increased the number of rods at 72 hpf (*sox11* MO: 20.3±11.1 rods/section; *sox11* MO + cyclopamine: 43.8±10.5 rods/section; p = 0.02; Figures 5C and S4B), and corrected the lamination and differentiation defects that were associated with colobomatous retinas (Figure S4C). In a reciprocal experiment, embryos were injected with half the full dose of each *sox11* MO, and treated with either a Hh agonist (purmorphamine) or vehicle control (DMSO) from 5.5–24 hpf. We used a sub-threshold dose of purmorphamine (75 µM), which did not cause coloboma when given alone (Figure 5B). In contrast, when the half dose of *sox11* MO was combined with purmorphamine, the prevalence of lens malformations at 24 hpf and coloboma at 2 dpf significantly increased (*sox11* MO half dose + purmorphamine: 57.9±10.2% malformed lens, 57.2±5.3% coloboma; *sox11* MO half dose + DMSO: 24.2±3.5% malformed lens, 28.9±5.9% coloboma; p<0.0001; Figures 5B and S4A). Together, these data demonstrate that deficiency of Sox11 increases Hh signaling, resulting in defects in ocular morphogenesis and reduced rod photoreceptor number.

Finally, we injected *sox11a* and *sox11b* mRNA into wildtype zebrafish embryos and evaluated the prevalence of a cyclopic phenotype, which is classically associated with reduced Hh pathway activity [3], at 24 hpf. Injection of *sox11a* and *sox11b* mRNA caused a cyclopic phenotype in 33.8±2.9% of the embryos, whereas only 4.2±0.7% of embryos had cyclopia when injected with a control *td*-*tomato* mRNA (p<0.001; Figure 5D). Taken together, these results demonstrate that Sox11 is required to limit Hh signaling during zebrafish ocular development.

### Transcription of *sonic hedgehog a* (*shha*) is strongly upregulated in *sox11* morphants

**Figure 6:**
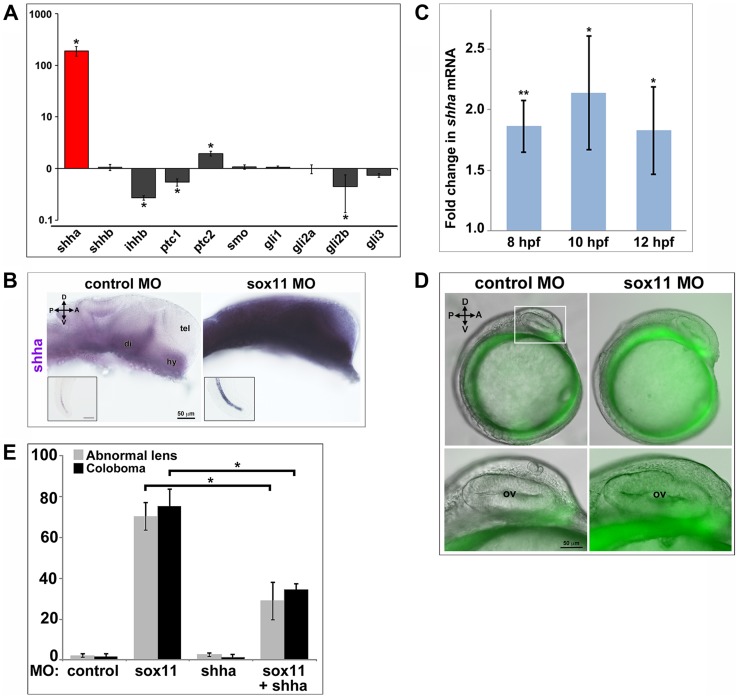
*Shha* expression is upregulated in *sox11* morphants. (A) QPCR performed on mRNA from control and *sox11* morphant heads at 24 hpf revealed a dramatic upregulation of *shha* expression, and a small but significant increase in *ptc2* expression, in *sox11* morphants compared to controls (n = 70 embryos per group, 3 independent biological replicates). Relative transcript abundance was normalized to *gapdh* levels. The Y-axis (log-scale) represents the mean ratio of *sox11* morphant to control expression for three biological and three technical replicates. *p<0.01, Student's *t*-test. (B) In situ hybridization with a *shha* probe on control (left) and *sox11* morphant (right) embryos at 24 hpf revealed expanded *shha* expression in *sox11* morphants throughout the brain and also in the notochord (inset). Numbers of embryos analyzed: n = 15 embryos per group, 3 independent repeats. (C) QPCR performed on mRNA from control and *sox11* morphant heads at 8, 10 and 12 hpf demonstrated an upregulation of *shha* expression in *sox11* morphants compared to controls. Relative transcript abundance was normalized to *gapdh* levels and is presented as the mean fold-change in expression relative to controls (n = 60 embryos per group, 3 independent biological repeats). **p<0.001, *p = 0.01, Student's *t*-test. (D) *Sox11* morphants (right) on the *ptc2:EGFP* background displayed elevated GFP expression in the midline at 12 hpf compared to control morphants (left). The bottom panels are an enlargement of the boxed area indicated in the top left panel. Number of embryos analyzed: control MO, n = 34; *sox11* MO, n = 41, 3 independent biological replicates. (E) Co-knockdown of *shha* and *sox11* reduced the proportion of embryos displaying abnormal lens and coloboma phenotypes at 24 hpf and 2 dpf, respectively. Number of embryos analyzed: 24 hpf control MO, n = 186; 2 dpf control MO, n = 165; 24 hpf *sox11* MO, n = 199, 2 dpf *sox11* MO, n = 182; 24 hpf *shha* MO, n = 249; 2 dpf *shha* MO, n = 231; 24 hpf *sox11*+ *shha* MO, n = 207; 2 dpf *sox11*+ *shha* MO, n = 190; 3 independent biological replicates. *p<0.0001, Student's *t-*test. D, dorsal; V, ventral; A, anterior; P, posterior; hpf, hours post fertilization; dpf, days post fertilization; R, retina; di; diencephalon, tel, telencephalon; hy, hypothalamus; MO, morpholino.

Zebrafish possess five Hedgehog ligands (Sonic hedgehog a and b, Indian hedgehog a and b, and Desert hedgehog), two Patched and one Smoothened receptor, and four Gli effectors. To determine whether expression of any of these pathway members was altered in *sox11* morphants, we performed qPCR on mRNA prepared from 18 and 24 hpf control and *sox11* morphant heads. At 18 hpf, no significant gene expression changes were observed, except for *gli2a* and *gli3*, which were both slightly elevated in *sox11* morphants (Figure S5A). In contrast, at 24 hpf we observed a very strong increase (189-fold) in the expression of *shha* in *sox11* morphants relative to controls, as well as a modest decrease in *ihhb*, *ptc1*, and *gli2b* expression, and a 2-fold increase in expression of *ptc2* (Figure 6A). The increase in *shha* expression in *sox11* morphants appeared to be dose-dependent, as injection of one-half the full dose of *sox11* MOs resulted in only a 65-fold elevation in *shha* (Figure S5B). In situ hybridization revealed greatly increased *shha* signal intensity in regions of the *sox11* morphant embryo that normally express *shha*, such as the ventral forebrain and the notochord (Figure 6B), with ectopic expression observed in the dorsal midbrain and telencephalon (Figure 6B). These results suggest that the ocular phenotypes in *sox11* morphants are caused by elevated levels of Shha. However, we were puzzled that *shha* transcript levels were not significantly increased at 18 hpf (Figure S5A), and yet cyclopamine treatment from 5.5–13 hpf rescued the ocular defects of *sox11* morphants. Therefore, we asked whether *shha* levels were elevated in *sox11* morphants at earlier time points. We performed qPCR analysis on mRNA from control and *sox11* morphants at 8, 10, and 12 hpf and found that *shha* levels are elevated approximately 2-fold in *sox11* morphants at these time points (Figure 6C). Moreover, using the *ptc2:GFP* line, we detected increased GFP levels in the ventral midline of *sox11* morphants at 12 hpf, confirming that Hh signaling was elevated at this stage (Figure 6D). Taken together, these results suggest that knockdown of *sox11* results in elevated expression of *shha*, and an increase in Hh signaling, as early as 8–12 hpf when the optic vesicle is evaginating from the midline.

To test the hypothesis that elevated Shha levels cause the ocular phenotypes of *sox11* morphants, we knocked down both *shha* and *sox11* simultaneously (using our *sox11* MOs and a previously described *shh*a MO [35]) and scored embryos at 24 hpf and 2 dpf for malformed lens and coloboma phenotypes, respectively. We used a low dose of the *shha* MO (3.14 ng/embryo), which by itself did not produce lens defects, coloboma, or rod photoreceptor defects (Figures 6E and S5C). The prevalence of ocular phenotypes was significantly reduced in the double morphants (*sox11* MO: 70.3%±6.7% malformed lens, 75.4%±8.3% coloboma; *sox11* + *shha* MOs: 28.9%±9.2% malformed lens; 34.7%±2.7% coloboma; p<0.0001; Figures 6E and S5C). Rod photoreceptor number was also significantly increased at 3 dpf in the double *shha*/*sox11* morphants, however it did not reach the levels observed in controls (*sox11* MO: 5.8±7.1 rods/section; *sox11* + *shha* MOs: 14.6±2.3 rods/section; p<0.05; Figure S5D). We performed qPCR analysis on *sox11* morphants treated with cyclopamine and purmorphamine and confirmed that these treatments caused a decrease and an increase in *shha* transcript levels, respectively (Figure S5E, F). Cyclopamine treatment of *sox11* morphants also restored expression of the Hh target gene *ptc2* to control levels (data not shown). Moreover, qPCR analysis of embryos injected with control or *sox11* mRNA confirmed that overexpression of *sox11* resulted in a concomitant decrease in *shha* expression (Figure S5G). Finally, we determined that there was not a reciprocal regulation of *sox11* by *shha*, because injection of the *shha* morpholino alone did not result in a change in expression of *sox11a* or *sox11b* (Figure S5H). Taken together, these results demonstrate that Sox11 controls levels of Hh signaling primarily through negative regulation of *shha* expression, and that limiting *shha* expression is essential for proper ocular morphogenesis.

### Bmp7b can rescue the ocular phenotypes in *sox11* morphants

**Figure 7:**
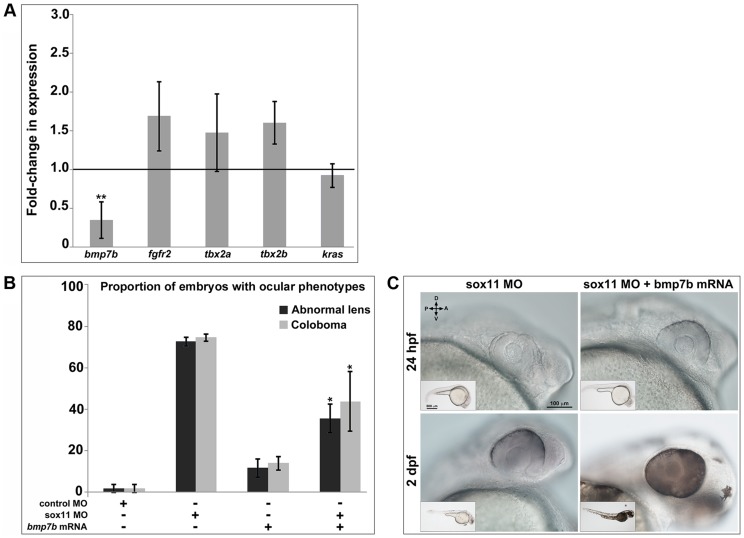
*Bmp7b* expression is reduced in *sox11* morphants. (A) QPCR was performed on mRNA from control and *sox11* morphant heads at 24 hpf for known repressors of *shha* transcription. A significant downregulation of *bmp7b* was observed in *sox11* morphants compared to controls. Relative transcript abundance was normalized to *gapdh* levels and is presented as the mean fold-change in expression relative to controls (n = 50 embryos per group, 3 independent biological repeats). **p<0.01, Student's *t* –test. (B) Injection of *bmp7b* mRNA significantly reduced the proportion of *sox11* morphants displaying abnormal lens and coloboma phenotypes at 24 hpf and 2 dpf, respectively. Number of embryos analyzed: 24 hpf control MO, n = 127; 2 dpf control MO, n = 123; 24 hpf *sox11* MO, n = 282; 2 dpf *sox11* MO, n = 274; 24 hpf *bmp7b* mRNA, n = 95; 2 dpf *bmp7b* mRNA, n = 91; 24 hpf *sox11* MO + *bmp7b* mRNA, n = 140, 2 dpf *sox11* MO + *bmp7b* mRNA, n = 134; 3 independent biological replicates. *p<0.006. (C) Brightfield images of a representative *sox11* morphant and a *sox11* morphant rescued with *bmp7b* mRNA, taken from the set of embryos analyzed in (B). D, dorsal; V, ventral; A, anterior; P, posterior; hpf, hours post fertilization; dpf, days post fertilization; MO, morpholino.

Thus far, our data strongly suggest that Sox11 is required to limit levels of *shha* expression during ocular development. However, Sox11 and other members of the SoxC family have previously been shown to function as transcriptional activators rather than repressors [36]–[38]. Furthermore, a scan of the *shha* promoter revealed no perfect consensus binding sequences for Sox factors (not shown; [37]), and the expression domains of *sox11* and *shha* only partially overlap in the ventral midline during ocular morphogenesis. Therefore, we hypothesized that Sox11 negatively regulates Shha indirectly and perhaps non-cell autonomously, by activating the expression of an upstream inhibitor of Shha. We searched the literature to identify candidate Shha repressors that are expressed in the forebrain during development, and then asked whether expression of any of these factors was reduced in *sox11* morphant heads at 24 hpf (Figure 7A). We analyzed five candidate genes: *bmp7b*, *fgfr2*, *tbx2a*, *tbx2b*, and *kras*, which had been shown previously to negatively regulate *shha* expression during development [39]–[44]. Of these five, *bmp7b* showed significantly decreased expression in *sox11* morphants compared to controls (Figure 7A). Bmp7b represents a good candidate intermediary between Sox11 and *shha* for several reasons. First, *Bmp7* null mice display microphthalmia and optic fissure defects, similar to *sox11* null mice [45]. Second, *bmp7* is expressed in the ventral midline and proximal optic vesicle in the mouse [45], and *bmp7b* is expressed in the forebrain adjacent to the optic vesicle in zebrafish at 18 hpf in a similar pattern to *sox11* [41]. Third, *bmp7* expression was reported to be reduced in *Sox11^−/−^* mice [13]. And finally, a scan of the *bmp7b* promoter revealed two perfect Sox consensus binding sites [37] located approximately 950 bp upstream of the transcription start site (not shown).

Because we had detected elevated *shha* levels as early as 8 hpf in *sox11* morphants, we asked whether *bmp7b* expression is also downregulated at that time. qPCR analysis revealed that *bmp7b* transcript levels were significantly reduced at 8, 10, and 12 hpf in *sox11* morphants when compared to controls (Figure S6). Interestingly, *bmp7b* expression increased to just above control levels at 18 hpf, before declining significantly again at 24 hpf. This rebound in *bmp7b* expression at 18 hpf precisely mirrors the normal levels of *shha* expression in *sox11* morphants at this time (Figure S5A). Taken together, these data suggest that the initial decrease in *bmp7b* expression (and corresponding elevation of *shha*) caused by knockdown of *sox11* induces a compensatory pathway that works to bring transcriptional levels back to normal, but that the continued knockdown of *sox11* results in renewed dysregulation of *bmp7b* and *shha*.

We reasoned that if Bmp7b functions downstream of Sox11 and upstream of Shha, then expression of *bmp7b* in *sox11* morphants should rescue the ocular phenotypes caused by elevated Hh signaling. To test this hypothesis, we injected *bmp7b* mRNA into control and *sox11* morphant embryos, and determined the proportion of embryos that displayed lens defects and coloboma at 24 hpf and 2 dpf, respectively. We found that co-injection of *bmp7b* mRNA into *sox11* morphants significantly reduced the number of embryos displaying ocular phenotypes (*sox11* MO: 72.6±2.22% malformed lens, 74.5±1.8% coloboma; *sox11* MO + *bmp7b* mRNA: 35.6±6.9% malformed lens; 43.8±14.4% coloboma; p<0.001; Figure 7B, C), although the rescue was not as large as that observed with cyclopamine treatment. These data suggest that Sox11 negatively regulates *shha* at least in part through Bmp7b.

### Sox4 can compensate for the loss of Sox11

As functional redundancy between SoxC family members has been observed in mouse models [10],[36],[46], we investigated whether another SoxC factor could compensate for the loss of Sox11 during zebrafish ocular morphogenesis. By in situ hybridization and qPCR, we observed elevated expression of the SoxC factor *sox4a* in *sox11* morphants at 24 and 36 hpf, suggesting that *sox11* deficiency induces a compensatory increase in *sox4* expression (Figure S7A, B). We then injected *sox4* mRNA into *sox11* morphants and found that this significantly reduced the proportion of embryos with lens and coloboma phenotypes (Figure S7C). This result suggests that increased Sox4 expression may buffer the effects of Sox11 deficiency. Consistent with this hypothesis, we observed a significantly greater proportion of embryos with coloboma in *sox4/sox11* double morphants than when either gene was knocked down alone (data not shown).

### Identification of *SOX11* variants in patients with coloboma

**Figure 8:**
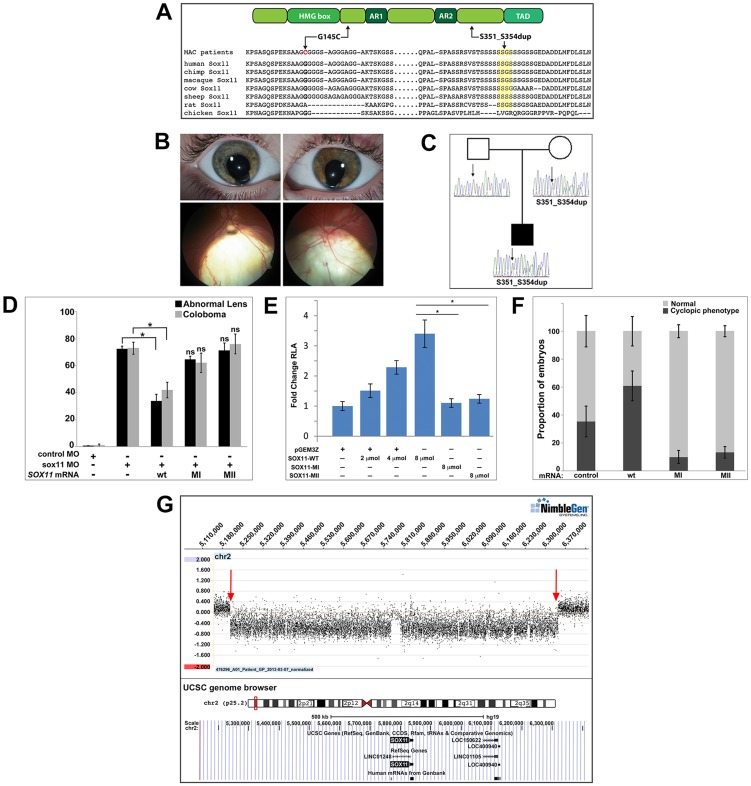
Association of *SOX11* locus with MAC. (A) Schematic representation of SOX11, indicating the positions of the two MAC sequence variants, and an alignment of the SOX11 protein sequence encompassing the two affected regions. (B) Photographs of the S351–354dup proband, indicating bi-lateral iris coloboma (top) and retino-choroidal coloboma (bottom). (C) Pedigree showing the S351–354dup proband and his parents. The proband's mother also carries the S351–354dup mutation, but does not have coloboma. (D) SOX11 mRNA containing G145C (MI) or S351–354dup (MII) did not rescue the abnormal lens or coloboma phenotypes of *sox11* morphants. Number of embryos analyzed: 24 hpf control MO, n = 202; 2 dpf control MO, n = 174; 24 hpf *sox11* MO, n = 148; 2 dpf *sox11* MO, n = 133; 24 hpf *sox11* MO +wild type *SOX11* mRNA, n = 177; 2 dpf *sox11* MO + wild type *SOX11* mRNA , n = 159; 24 hpf *sox11* MO + MI *SOX11* mRNA, n = 203, 2 dpf *sox11* MO + MI *SOX11* mRNA, n = 188; 24 hpf *sox11* MO + MII *SOX11* mRNA, n = 219, 2 dpf *sox11* MO + MII *SOX11* mRNA, n = 201; average of three independent biological replicates. *p<0.0001, Fisher's exact test. (E) GDF5-luciferase reporter activity. Transfection of either SOX11 G145C (MI) or S351–354dup (MII) did not significantly enhance luciferase levels. Firefly luciferase activity was normalized to *Renilla* luciferase and is represented as mean fold change over the empty vector (pGEM3Z) from three biological and six technical replicates (*p<0.0001, Student's *t*-test). (F) Whereas overexpression of wild type (WT) human *SOX11* mRNA increased the proportion of embryos with a cyclopic phenotype compared to injection of control (*td-Tomato*) mRNA, human *SOX11* mRNA containing G145C (MI) or S351–354dup (MII) did not cause cyclopia. Number of embryos analyzed: control mRNA, n = 67; WT *SOX11* mRNA, n = 62; MI *SOX11* mRNA, n = 165; MII *SOX11* mRNA, n = 128, 3 independent biological repeats. *p<0.006 (G) Array CGH data for 2p25.2 demonstrating deletion breakpoints (red arrows) in a patient with agenesis of the optic nerve, microphthalmia, and developmental delay. The corresponding annotated genomic region, modified from the UCSC genome browser (http://genome.ucsc.edu/), is shown below; *SOX11* is the only protein-coding gene within the deleted region. MAC, microphthalmia, anophthalmia, and coloboma; MO, morpholino.

To investigate whether *SOX11* mutations contribute to patient phenotypes, the coding region was sequenced in DNA samples from 79 MAC patients [47]. These DNA samples had been previously screened for mutations in two other coloboma-related genes, *GDF3* and *GDF6* [47]–[49]. We identified heterozygous sequence changes in two probands (Figure 8A), both of whom are Canadians of white European ancestry. The first, a c.488G→T missense mutation in a coloboma patient, is predicted to result in a G145C amino acid alteration, considered damaging by SIFT analysis (http://sift.jcvi.org/). The second variant, a 12-nucleotide duplication (c.1106–1117) in a patient with bilateral iris and retino-choroidal coloboma (Figure 8B), is predicted to result in an in-frame, four amino acid duplication (S351–354dup). The affected amino acid residues are located outside previously defined functional domains and are conserved in chimp and macaque SOX11 (Figure 8A). These variants were absent from dbSNP and the 1000 Genomes databases, and from the NHLBI database comprising more than ten thousand exomes (Figure S8A). Sequencing of *SOX11* from the probands' family members revealed that the S351–354dup alteration was present in the proband's mother, who did not exhibit a phenotype clinically (Figure 8C). In light of the rod photoreceptor phenotype in zebrafish *sox11* morphants, an electroretinogram (ERG) was performed on the mother carrying the S351–354dup alteration. This analysis demonstrated a reduction in scotopic b-wave amplitude, indicating reduced rod photoreceptor function (Figure S8B). In addition, her 10Hz dim white flicker response was appreciably reduced, and was associated with a change in latency. The mother was asymptomatic at the time the ERG was performed, which may reflect her young age (37 years). Her cone flicker response was normal.

Intrigued by the presence of phenotypic effects in a heterozygote only on targeted testing, 384 DNA samples derived from patients undergoing screening for hemochromatosis were sequenced, which detected the S351–354dup variant in three individuals, whilst the G145C variant was absent. Unfortunately, these three carriers could not be recalled for clinical examination.

To determine whether the two *SOX11* sequence variants had functional consequences, their ability to rescue the lens and coloboma phenotypes of zebrafish *sox11* morphants was compared to wild type human *SOX11* mRNA. Whereas wild type *SOX11* mRNA significantly reduced the proportion of *sox11* morphants displaying lens defects and coloboma, no significant rescue was observed with mRNA containing either *SOX11* variant (Figures 8D and S8D), suggesting that both sequence changes compromise SOX11 function. Next, we utilized a luciferase reporter containing the promoter region of the SOX11 target gene *GDF5* [50] to further analyze the functional consequences of the two mutations. Expression of increasing amounts of wild type *SOX11* in COS-7 cells produced a dose-dependent increase in luciferase activity from the *GDF5* reporter (Figure 8E). In contrast, transfection of equivalent amounts of either *SOX11* variant did not enhance luciferase activity over the empty vector control (Figure 8E), although the variants showed comparable levels of protein expression by Western blot (Figure S8C). Equivalent results were obtained with the luciferase assay in two additional cell lines (HEK293 and HeLa; data not shown). To further confirm that the two *SOX11* sequence variants are functionally compromised, we overexpressed them in zebrafish and quantified the proportion of embryos that exhibited a cyclopic phenotype at 24 hpf. Whereas injection of WT human *SOX11* mRNA caused a significant increase in the proportion of cyclopic embryos compared to injection of control td-Tomato mRNA (61.16±10.7% in WT *SOX11* injected vs. 35.3±11% in control injected; p<0.05), neither of the *SOX11* sequence variants produced elevated levels of cyclopia (G145C, 10.0±4.8%, S351–354dup, 13.1±4.11%; Figures 8F and S8E). Taken together, these data suggest that the two variants compromise SOX11's transactivation ability.

Finally, array comparative genomic hybridization (array CGH) was performed on DNA from a patient with microphthalmia, unilateral optic nerve agenesis, and a *de novo* chromosome 2p25 deletion [18]. This defined a 1.14 Mb segmental deletion (5,206,155–6,343,906; chromosome build GRCh37), encompassing an interval within which *SOX11* is the only protein-coding gene (Figures 8G and S8F). Taken together, these data demonstrate that perturbed SOX11 function, either through mutation or decreased gene dosage, contributes to structural (microphthalmia/coloboma) or functional (rod photoreceptor) phenotypes.

## Discussion

This study reveals a novel role for Sox11 in maintaining the correct level of Hedgehog (Hh) signaling during ocular morphogenesis. We demonstrate that knockdown of Sox11 in zebrafish perturbs lens formation, induces coloboma, and reduces the number of differentiated rod photoreceptors – phenotypes that can be rescued by pharmacological inhibition of the Hh pathway (cyclopamine) or morpholino inhibition of *shha*. Comparable lenticular and coloboma phenotypes have also been observed in murine mutants [13], demonstrating that Sox11's function in vertebrate ocular development is evolutionarily conserved. However, the perinatal lethality of *Sox11* null mice has precluded a thorough in vivo assessment of rod photoreceptor differentiation, which mostly occurs postnatally. Expression of the rod photoreceptor genes *Nrl*, *Nr2e3*, and *Sag* (Rod arrestin) is significantly reduced in E16 retinas from *Sox11^−/−^* mice [51], suggesting that Sox11 does regulate aspects of rod photoreceptor differentiation in mammals. However, in retinal explants derived from *Sox11* null mice and cultured for several days, reduced rod photoreceptor number was not observed [51]. Our data suggesting that early, midline-derived Shh influences rod photoreceptor differentiation (see below), indicates that retinal explants, being removed from the source of extra-retinal Shh, may not accurately reflect the in vivo response of retinal progenitor cells to their environment. In this context, the external embryogenesis, rapid pace of retinal development, and continual rod photoreceptor genesis in the zebrafish have benefitted our studies and permitted us to uncover for the first time both the mechanism of Sox11's action during early ocular development, as well as a role for Sox11 in regulating rod photoreceptor differentiation.

A second key finding of our study is that Sox11 acts upstream of Hh signaling specifically by negatively regulating transcription of the ligand *shha*. In Sox11-deficient embryos, we observed a strong increase in *shha* expression in the ventral forebrain, as well as an expansion of the *shha* territory into the dorsal diencephalon and the telencephalon. Therefore, in addition to regulating expression of *shha* expression in the ventral midline, our data suggest that Sox11 is also required to prevent activation of *shha* in the more dorsal regions of the brain. Within the retina, the expression of *sox11a* in the GCL at 48 hpf suggests that Sox11 continues to regulate Hh signaling during retinal neurogenesis.

The magnitude of the increase in *shha* expression in the absence of *sox11* (over 180-fold) at 24 hpf suggests that loss of *shha* transcriptional repression is accompanied by a significant positive transcriptional feedback loop. However, the Hh target gene *ptc2* demonstrated a much smaller increase in expression (2-fold) at this time, raising the question of why the dramatic upregulation in *shha* did not produce a correspondingly large transcriptional response. One possible explanation is that post-transcriptional mechanisms narrow the range of Shha protein expression in *sox11* morphants. Moreover, additional feedback mechanisms may work to attenuate the transcriptional response of Hh target genes such as *patched*. In any case, the elevated and expanded GFP expression in the Hh reporter line *ptc2:GFP*, as well as the rescue by cyclopamine and *shha* co-knockdown, strongly argue that the rise in *shha* transcription induced by *sox11* deficiency has functional consequences.

In the absence of Sox11, we observed an early expansion of the optic stalk marker *pax2.1* in the optic vesicle, and a later reduction in the retinal marker *pax6a*. Such altered proximodistal patterning of the optic vesicle has been observed in several models of elevated Hh signaling [4], [5], [25], [52], [53]. The increased apoptosis in the lens and its abnormal development may be attributable to reduced *pax6a* expression in *sox11* morphants, since similar phenotypes were observed in a lens-specific *Pax6* conditional mutant mouse model [54]. In parallel, we suggest the expansion of *pax2.1* expression due to elevated levels of Shh enlarged the area of the optic vesicle that was specified as optic stalk, hindering closure of the choroid fissure and thus causing coloboma. Elevated Hh signaling could also account for the increase in mitotic cells in the retina, as this pathway is known to be mitogenic [55].

Previous studies in zebrafish have shown that blocking early Hh signaling, either with *shha* and *shhb* morpholinos or by cyclopamine treatment, caused a reduction in rhodopsin expression in the retina, suggesting that Hh signaling promotes rod photoreceptor differentiation [56]. However, murine studies have found that activation of the Hh pathway results in a non-cell autonomous inhibition of rhodopsin expression [57], which is consistent with our results. Moreover, loss of Shh was shown to cause accelerated differentiation of rods and cones in a conditional mouse model [58]. The seemingly paradoxical response to increased and decreased Shh levels is potentially explained by the requirement for precise Shh dosage, with either alteration resulting in reduced photoreceptor number. This accords with a comparable model for Shh's effect on reactive astrocytes [59], and is a well-recognized feature of transcription factors, as exemplified by the effects of altered *Pax6* dosage in inducing microphthalmia [60].

Interestingly, we observed a significant increase in *shha* expression at 8–12 hpf, when the optic vesicle is evaginating from the midline, and we confirmed that Hh signaling was increased at this time using a *ptc2:GFP* reporter line (Figure 6). Furthermore, treatment of *sox11* morphants with cyclopamine during this developmental window was sufficient to restore rod photoreceptor number at 72 hpf. Thus, taken together, these data indicate that early, midline-derived Shh influences rod photoreceptor differentiation. This is not the first demonstration that early midline Hh signals influence later neurogenesis in the retina. It has been shown previously that the timely progression of *ath5* expression in the retina, which coincides with the activation of neurogenesis, depends on axial Shh [61]. As *ath5*-positive cells contribute significantly to the rod photoreceptor lineage [62], it is plausible that elevated Shh coming from the midline in *sox11* morphants delays rod photoreceptor differentiation by influencing the cell-intrinsic neurogenic program of retinal progenitor cells.

Although the phenotypes of zebrafish *blowout* (*blw*) mutants and *sox11* morphants are similar with respect to coloboma, *blw* mutants do not appear to have a defect in the differentiation of rod photoreceptors or any other retinal cell types. This is surprising, given the well-described influence of Hh signaling on retinal neurogenesis [3], [4], [33], [56], [61], [63]–[66]. Moreover, patients with elevated Hh signaling due to heterozygous loss of function mutations in *PTCH* exhibit retinal abnormalities, and *PtchlacZ^+/−^* mice display a delay in photoreceptor and horizontal cell maturation at P5, all of which is consistent with our data [67]. One possible explanation as to why *sox11* morphants and *blw* mutants differ in this aspect of their phenotype is that the mutation in *ptc2* may be a partial loss of function allele, which is supported by the observation that *ptc2* morphants display more severe phenotypes than *blw* mutants [25].

Since SoxC factors are generally considered to function as transcriptional activators rather than repressors [36], [38], we hypothesized that Sox11 regulates Shha indirectly, through the induction of a repressor. Indeed, we found that *bmp7b* expression was significantly reduced in *sox11* morphants, and that injection of *bmp7b* mRNA into *sox11* morphants could rescue the lens and coloboma phenotypes (Figure 7). As Bmp7 has previously been shown to antagonize Shh signaling [42], [68], our results are compatible with a model whereby Bmp7 functions downstream of Sox11 to limit Shh expression during ocular morphogenesis. However, since the magnitude of the *bmp7b* rescue was not as large as that observed with cyclopamine treatment, additional mechanisms linking Sox11 with the regulation of Hh signaling are likely.

So far, more than 27 genes are associated with coloboma in humans [2], however mutations in these account for less than 20% of cases. Consequently, it is important to define additional causative genes, both to extend understanding of pathogenesis and define pathways that may be amenable to therapeutic modulation. Our work, in combination with previous studies [13], strongly supports a contribution from SOX11 to coloboma phenotypes, however our data indicate that the relationship is complex. With a 50% reduction in gene dosage (2p25 segmental deletion; Figure 8G), a profound phenotype was observed. In contrast, milder coding changes (S351–354dup) with a low prevalence in the general population, resulted in incompletely penetrant phenotypes, with the unaffected carrier exhibiting a sub-clinical phenotype, only detectable on ERG testing. Since the variants had significantly reduced function on *in vitro* and *in vivo* assays, this suggests that such mild alleles contribute to MAC but may be insufficient to induce phenotypes alone in all cases. Coloboma, like many developmental defects, exhibits extensive phenotypic variability, suggesting complex relationships between disease genes and modifying alleles that complicate simple genotype-phenotype correlations. It is also possible that oligogenic inheritance is a factor in coloboma, in which individuals in non-penetrant families carry a combination of pathogenic alleles at two or more disease loci, as has been described for other genetically heterogeneous developmental disorders such as the ciliopathies [69]. Furthermore, functional redundancy between Sox subgroup family members is also commonly observed [36], [46], [70], suggesting that one SoxC family member may buffer the effects of mutation in a second. Consistent with this model, we observed elevated expression of the SoxC factor *sox4* in *sox11* morphants at 24 and 36 hpf, and found that the lens and coloboma phenotypes of *sox11* morphants could be rescued by injection of *sox4* mRNA (Figure S7). Finally, in light of the incompletely penetrant phenotypes evident with multiple other MAC-causing genes [47], [49], [71]–[73], a similar additive contribution from other SOX gene variants is highly plausible.

In summary, we describe here a novel role for Sox11 in regulating levels of Shh during ocular morphogenesis. It will be interesting to determine whether dysregulated Hh signaling underlies any of the additional developmental defects observed in *Sox11^−/−^* mice, such as congenital cardiac malformations and craniofacial anomalies. Future studies will continue to explore the mechanisms of how Sox11 regulates Hh signaling and Shh transcription, as well as the identification of direct molecular targets of Sox11 transcriptional control.

## Materials and Methods

### Zebrafish

The Tg (XlRho:EGFP)^fl1^ transgenic line has been previously described [28], and was generously provided by J.M. Fadool (Florida State University, Tallahassee, FL). The Tg (gfap:GFP)mi2001 line has been previously described [74] and was obtained from the Zebrafish International Resource Center (Eugene, OR). The Tg (3.2TαC-EGFP) line has been previously described [75], and was generously provided by S.E. Brockerhoff (University of Washington, Seattle, WA). *Tg*(*GBS-ptch2:nlsEGFP*) has been previously described [34] and was kindly provided by R. Karlstrom (University of Massachusetts, Amherst, MA). Zebrafish (*Danio rerio*) were reared, bred, and staged according to standard protocols [76], [77]. All animal procedures were carried out in accordance with the policies established by the University of Kentucky Institutional Animal Care and Use Committee (IUCAC).

### Morpholino (MO) injection and analysis

Morpholinos (MOs) were obtained from Gene Tools, LLC (Philomath, OR) and were prepared and injected as previously described [78]. The following MOs were used in this study: standard control MO, 5′-CCTCTTACCTCAGTTACAATTTATA-3′; *sox11a* MO1, 5′ –GTGCGTTGTCAGTCCAAAATATCAA-3′; *sox11b* MO1, 5′ –CATGTTCAAACACACTTTTCCCTCT; *shha*-MO: 5′CAGCACTCTCGTCAAAAGCCGCATT [35]. The specificity of the *sox11* morphant phenotype was confirmed using a second *sox11* morpholino placed further downstream of the first set (completely non-overlapping with *sox11a* MO1, and overlapping by only 4 nucleotides with *sox11b* MO1). Because the target site for this morpholino extended into the coding region (which is highly similar in sequence for both genes) it simultaneously targets both *sox11a* and *sox11b* (*sox11* MO2, 5′ –TCCGTTTGCPGCACCATG-3′; the “P” indicates a photo-cleavable moiety that was not used in this study). The *sox11* MO2 produced the same coloboma phenotype as the first set of MOs (Figure S1C). All data presented in this study are from embryos injected with *sox11a* MO1 and *sox11b* MO1. Unless stated otherwise, embryos were injected with 4.18 ng each of *sox11a* MO1 and *sox11b* MO1, 4.18 ng of the standard control MO, or with 3.14 ng of *shha* MO. We also confirmed that no abnormal phenotypes were observed when embryos were injected with 8 ng of standard control MO. To determine the efficiency of the *sox11* MOs, PCR fragments corresponding to the 5′UTRs of *sox11a* and *sox11b* encompassing the morpholino target sequences were amplified (using primers listed in Table S1) and cloned upstream and in frame with the EGFP gene in the pEF1α:GFP plasmid (Addgene plasmid 11154). One-cell stage zebrafish embryos were injected with 100 pg/embryo of pEF1α:GFP plasmid containing the MO binding site in the presence or absence of the *sox11* MOs. GFP expression in injected embryos was analyzed by fluorescence microscopy at 24 hpf.

### mRNA synthesis and injection

Zebrafish *sox11a* and *sox11b* or human wild type and variant *SOX11* coding sequences were PCR amplified (using primers listed in Table S1) and cloned into the pGEMT-easy vector (Promega). The pCRII-*bmp7b* plasmid has been previously described [41] and was a kind gift from Dr. S. Fabrizio (The Novartis Institutes for Biomedical Research, Cambridge, MA). The constructs were linearized and mRNA was prepared using the mMESSAGE mMACHINE kit (Ambion) according to manufacturer's instructions. Zebrafish *sox11a* and *sox11b* mRNAs (1.0 ng each), human *SOX11* mRNA (0.3 ng), zebrafish *bmp7b* mRNA (1.0 ng) or zebrafish *sox4a* and *sox4b* (0.5 ng each) were injected into zebrafish embryos at the one-cell stage. For mRNA rescue experiments, the mRNAs were either co-injected with *sox11* MOs, or were injected sequentially after injection of the MOs. As both methods produced similar results, the data presented here are for co-injection of mRNA and morpholino. Injections were always performed in triplicate, and a minimum of 55 injected embryos were analyzed in each experiment. For mRNA overexpression experiments, embryos were injected with either a control (*tdTomato*) mRNA, zebrafish *sox11a/b* mRNA, or human WT, G145C (MI), or S351–354dup (MII) *SOX11* mRNA, all at equimolar concentrations. The control mRNA was synthesized from pRSET-B-td-Tomato (kindly provided by Dr. D.A. Harrison, University of Kentucky, Lexington, KY). To compare control versus *sox11a/b* mRNA, 0.003 pmol of each mRNA was injected. To compare control versus human WT and variant *SOX11* mRNA, 0.0133 pmol of each mRNA was injected. Zebrafish embryos were injected at the one-cell stage, and embryos were scored for cyclopic phenotypes (one single eye in the center of the head, two eyes that were almost fused at the midline, or one normal eye and one vestigial eye) at 24 hpf.

### Patient analysis

To screen for mutations in human *SOX11*, PCR was performed using three sets of overlapping primers that spanned the entire coding region of the single-exon *SOX11* gene. The amplicons were sequenced on an ABI Prism 3100 capillary sequencer (Applied Biosystems), analyzed using DNABaser v.3.1.5 and sequence alignments were performed using ClustalW. Mutations were confirmed by bi-directional Sanger sequencing and RFLP analysis of the *SOX11* amplicons. Half of the 384 control DNA samples were screened by RFLP analysis, using TseI (NEB) for the G145C variant and SfcI (NEB) for the S351–S354dup variant, and the other half were screened by direct Sanger sequencing of the *SOX11* coding region. Array CGH analysis was performed using a custom designed Nimblegen 4×72 whole human genome array. Oligonucleotide probes were spaced approximately every 75 bp across a 2.65 Mb region at 2p25.2, and backbone probes covered the rest of the genome. Four technical replicates were performed on the proband's DNA, and two replicate hybridizations were performed for each parental DNA sample. Array hybridization and scanning were performed by the Roy Carver Center for Genomics at the University of Iowa (Iowa City, IA). Array data were analyzed using the segMNT analysis program (Nimblegen). Informed consent was obtained from all participants. Study approval was provided by the University of Alberta Hospital Health Research Ethics Board and the Ethics Committee of the IRCCS Oasi Maria SS Onlus, Troina, Italy.

### Pharmacological manipulations

Cyclopamine (Sigma) was resuspended at 1 mM concentration in 100% ethanol and diluted in fish water for exposure. A dose response curve was generated by exposing wild type embryos to 0.5, 1.0, and 2.0 µM of cyclopamine from 5.5–13 hpf, and the dose (2.0 µM) at which no abnormal phenotype and negligible toxicity was observed was used for control and *sox11* morphants. Purmorphamine (Calbiochem) was resuspended at 50 mM concentration in DMSO and diluted in fish water for exposures. Wild type embryos were exposed to 10–100 µM of purmorphamine from 5.5–24 hpf, and the dose (75 µM) at which no ocular phenotypes were observed was used to treat control and *sox11* morphants.

### Whole mount *in situ* hybridization, two-color fluorescent in situ hybridization (FISH) and immunohistochemistry

Whole mount in situ hybridization (WISH) and immunohistochemistry were performed essentially as previously described [78]. For FISH embryos were manually dechorionated and fixed in 4% paraformaldehyde (PFA) made with diethyl pyrocarbonate (DEPC)-treated PBS at 4°C overnight. The fixed embryos were sequentially cryoprotected in 10% sucrose-DEPC and 30% sucrose-DEPC at 4°C overnight. Embryos were then embedded in OCT (Ted Pella, Redding, CA) and frozen at −80°C. Ten-micron sections were collected using a cryostat (Leica CM1900, Leica Biosystems, Buffalo Grove, IL), placed on Superfrost plus glass slides (Fisher Scientific, Waltham, MA) and air dried at room temperature overnight. The sections were post-fixed in 1% PFA-DEPC and rehydrated in PBST-DEPC. The sections were permeabilized for 10 minutes with 1 µg/ml proteinase K. Sections were acetylated in triethanolamine buffer plus 0.25% acetic anhydride (Sigma-Aldrich, Saint Louis, MO), and then rinsed in DEPC treated water. Sections were hybridized with digoxigenin (DIG) and fluorescein (FITC) labeled probes (2.5 ng/µl) in hybridization buffer (0.25% SDS, 10% dextran sulfate, 1× Denhardt's solution , 200 µg/ml torula yeast tRNA, 50% de-ionized formamide, 1 mM EDTA, 600 mM NaCl, and 10 mM Tris pH 7.5 in DEPC-treated water) at 65°C in a sealed humidified chamber for a minimum of 16 hours. Following hybridization, the slides were rinsed in 5× SSC and then with pre-warmed 1× SSC/50% formamide. Endogenous peroxidase activity was quenched with 1% H_2_O_2_ for 30 minutes. Sections were blocked using 0.5% PE blocking solution (Perkin Elmer Inc, Waltham, MA) for at least 1 hour. For two-color FISH, sections were incubated first with anti-DIG-POD Fab fragment (Roche, Indianapolis, IN) at 4°C overnight. Subsequently, probe signal was detected using the TSA plus Cy3 kit (Perkin Elmer Inc, Waltham, MA) following the manufacturer's instructions. For the second color detection, the sections were treated with 1% H_2_O_2_ for 30 minutes and then incubated with anti-FITC-POD Fab fragment (Roche, Indianapolis, IN) at 4°C overnight. Subsequently, the FITC-labeled probe signal was revealed using TSA plus Fluorescein (Perkin Elmer Inc, Waltham, MA). Finally, sections were counterstained with 4′, 6-diamidino-2-phenylindole (DAPI; Sigma-Aldrich, Saint Louis, MO), mounted in 40% glycerol, and imaged on an inverted fluorescent microscope (Nikon Eclipse Ti-U; Nikon Instruments, Melville, NY) using a 40× objective.

The *sox11a*, *sox11b* and *shha* cDNAs were amplified (using primers listed in Table S1) and cloned from 48 hpf whole embryo cDNA. The *sox11b* and *NeuroD* antisense probes have been previously described [14]. The *pax6a* , *crx*, and *nr2e3* probes have been previously described, and were kindly provided by Y.F. Leung (Purdue University, Indiana). The *pax2.1* probe has been described previously [25] and was a gift from J.M. Gross (University of Texas, Austin, TX). The following primary antibodies and dilutions were used: Zpr-1 (1∶20; ZIRC), which labels red-green cones; Zn-8 (1∶10; ZIRC), which labels ganglion cells; anti-Prox-1 (1∶2000; Millipore), which recognizes horizontal cells; anti-PH3 (1∶500; Millipore), which marks cells in G2/M phase; 5E11 (1∶10; J.M. Fadool, Florida State University), which labels amacrine cells; and anti-PKCα (1∶300; Santa Cruz Biotechnology), which labels bipolar cells. Alexa Fluor secondary antibodies (Molecular Probes, Invitrogen) and Cy-conjugated secondary antibodies (Jackson ImmunoResearch) were all used at 1∶200 dilution. Sections from the same region of the eye were analyzed for quantification purposes. One section was quantified per individual embryo (for both control and *sox11* morphants).

### TUNEL Assay

Terminal deoxynucleotide transferase (TdT)-mediated dUTP nick end labeling (TUNEL) was performed on retinal cryosections using the ApopTag Fluorescein Direct In Situ Apoptosis Detection Kit (Millipore) according to the manufacturer's instructions. Sections from the same region of the eye were analyzed for quantification purposes. One section was quantified per individual embryo (for both control and *sox11* morphants).

### Real-time quantitative RT-PCR

RNA extracted from the heads of control, *sox11*, and *shha* morphant embryos at various time points was used to perform first-strand cDNA synthesis (GoScript Reverse Transcriptase System; Promega). Real time PCR was performed using either Maxima SYBR Green qPCR master mix (Thermo Scientific) or FastStart SYBR Green Master (Roche) on an iCycler iQ Real Time PCR Detection system (Bio-Rad) or LightCycler 96 (Roche) with primers listed in Table S1. Three biological replicates were performed for each experiment. The gene expression change was determined using a relative standard curve quantification method with *gapdh*, *atp5h*, or 18s rRNA [79] expression as the normalization control.

### Statistics

Statistical analysis was performed on all data using the GraphPad Prism 6.02 software. Continuous data were analyzed using Student's-*t*-test and Fisher's exact test. For all graphs, data are represented as the mean ± the standard deviation (s.d.).

### Dual luciferase assays

COS-7 cells were transfected with the pcDNA3 expression vector (Invitrogen) containing the coding region of wild type, G145C, or S351–354dup *SOX11*; the pGL3 Firefly Luciferase reporter vector (Promega) containing the *GDF5* core promoter was a kind gift from Akinori Kan (Harvard Medical School, Boston, MA) [80]; and the pRL-TK vector (Promega) containing *Renilla* luciferase driven by a ubiquitous tyrosine kinase promoter to control for transfection efficiency. Transfections were performed using Fugene 6 (Promega), following manufacturer's instructions. The total mass of DNA and molar ratios of pGL3 and pRL-TK were held constant across transfections, which were repeated a minimum of 6 times. Dose response curves were generated using wild type *SOX11* at 0∶100, 1∶20, 1∶10, and 1∶5 molar ratios to the *GDF5* reporter. The mutant *SOX11* variants were transfected at a 1∶5 molar ratio to the *GDF5* reporter. Firefly and *Renilla* luciferase activity were measured 24–36 hours post transfection using the DualGlo Luciferase Assay System (Promega). Data was analyzed as follows: Firefly luciferase (FFLuc) was baselined against untransfected control (UTC) samples ( = FFLuc – UTC) and normalized using the *Renilla* luciferase (RLuc). The Relative Luciferase Activity (RLA) was calculated as (FFLuc-UTC)/RLuc and compared between experimental and control transfections.

## Supporting Information

Figure S1 Efficiency and specificity of *sox11* morpholinos. (A) Schematic representation of the pEF1α:GFP plasmid containing a portion of the *sox11* 5′ UTR placed upstream of the GFP reporter (top). The binding site for the *sox11* morpholino is shown in red. Separate reporters were constructed for the *sox11a* and *sox11b* MOs. (Center) Lateral view (anterior at top) of 24 hpf embryos injected with EF1α- *sox11a/b*-GFP plasmids alone (left) or with both *sox11* MOs. No GFP expression was detected in the embryo injected with *sox11* MOs. (Bottom) Quantification of the proportion of GFP-positive embryos at 24 hpf. The *sox11* MOs were highly effective at blocking GFP expression. Number of embryos analyzed: pEF1α-*sox11*-GFP plasmid alone, n = 169; pEF1α-*sox11*-GFP + *sox11* MOs, n = 140, 3 independent repeats; *p = 0.004, Student's *t*-test. (B) Both *sox11a* and *sox11b* contribute to abnormal lens and coloboma phenotypes observed in *sox11* morphants. The proportion of embryos displaying either phenotype was significantly higher when injected with *sox11a* and *sox11b* MOs simultaneously, compared to either MO alone. Number of embryos analyzed: 24 hpf control MO, n = 463; 2 dpf control MO, n = 441; 24 hpf *sox11a* MO, n = 229; 2 dpf *sox11a* MO, n = 214; 24 hpf *sox11b* MO, n = 341; 2 dpf *sox11b* MO, n = 316; 24 hpf *sox11a* + *sox11b* MO, n = 271; 2 dpf *sox11a* + *sox11b* MO, n = 262, 3 independent repeats. *p<0.001, Fisher's exact test. (C) A second non-overlapping *sox11* MO (that targeted both *sox11a* and *sox11b* simultaneously) produced the same coloboma phenotype in similar proportion to the first set. Number of embryos analyzed: control MO, n = 186 embryos; *sox11* MO, n = 194, 3 independent repeats; *p<0.001, Fisher's exact test. MO, morpholino; hpf, hours post fertilization; dpf, days post fertilization.
(TIF)

Figure S2 Cell proliferation and apoptosis in *sox11* morphants. (A) Quantification of TUNEL+ cells in the optic vesicle, lens, and retina of control and *sox11* and morphants from 18–72 hpf. *Sox11* morphants had an elevated number of TUNEL+ cells in the optic vesicle at 18 hpf. Additionally, *sox11* morphants consistently displayed more TUNEL+ cells in the anterior lens compared to controls from 24 -72 hpf. Number of embryos analyzed: 18 hpf control MO, n = 20; 18 hpf *sox11* MO, n = 22; 24 hpf control MO, n = 15; 24 hpf *sox11* MO, n = 19; 48 hpf control MO, n = 10; 48 hpf *sox11* MO, n = 13; 72 hpf control MO, n = 12; 72 hpf *sox11* MO, n = 12; average of 3 independent biological replicates. **p<0.00001, *p<0.01, Student's *t*-test. (B) Representative transverse sections of control (left column) and *sox11* (right column) morphants at 18, 24, and 48 hpf, taken from the set of individuals analyzed in (A). At 48 hpf, TUNEL+ cells were detected within the colobomatous tissue and the region of the optic stalk in *sox11* morphants (arrow, bottom right). (C) *Sox11* morphant retinas had more PH3^+^ cells than controls from 18–72 hpf. Number of embryos analyzed: 18 hpf control MO, n = 12; 18 hpf *sox11* MO, n = 15; 24 hpf control MO, n = 20; 24 hpf *sox11* MO, n = 19; 48 hpf control MO, n = 10; 48 hpf *sox11* MO, n = 12; 72 hpf control MO, n = 14; 72 hpf *sox11* MO, n = 12; average of 3 independent biological replicates. **p<0.001, *p<0.01, Student's *t*-test. (D) Representative transverse sections of control (left column) and *sox11* (right column) morphants at 18, 24, and 48 hpf, taken from the set of individuals analyzed in (C). D, dorsal; V, ventral; MO, morpholino; hpf, hours post fertilization; ON; optic nerve; OV, optic vesicle; R, retina; L, lens.
(TIF)

Figure S3 Retinal neurogenesis in *sox11* morphants. (A) Retinal cell types were visualized by immunohistochemistry (ganglion, amacrine, horizontal, and bipolar cells) or with fluorescent reporter transgenic lines (Tg(gfap:GFP)mi2001 for Müller glia and Tg(3.2TαC-EGFP) for cones) in controls (left) and *sox11* morphants (center, right) at 3 dpf. In *sox11* morphants without coloboma (center), the retinas are well laminated and had normal numbers of ganglion, amacrine, horizontal, and bipolar cells, cone photoreceptors, and Müller glia. However, *sox11* morphants with coloboma (asterisk; right) had poorly laminated retinas and reduced numbers of differentiated retinal cell types, indicating delayed retinal development. (B) Quantification of numbers of late-born retinal cell types in control and *sox11* morphants without coloboma. Only rod photoreceptors displayed a significant reduction. Number of embryos analyzed: control MO, n = 19; *sox11* MO without coloboma, n = 25, 3 independent repeats.**p<0.00001; ns = p>0.05, Student's *t*-test.(C) At 4 dpf, *sox11* morphants have more mature rod photoreceptors than at 3 dpf but the number remains significantly less than controls (*p<0.001, Student's *t*-test); MO, morpholino; dpf; days post fertilization; L, lens; GCL, ganglion cell layer; INL, inner nuclear layer; ONL, outer nuclear layer; ON, optic nerve.
(TIF)

Figure S4 Elevated Hh signaling contributes to the abnormal ocular phenotypes displayed by *sox11* morphants. (A) Representative brightfield images of *sox11* morphants treated with cyclopamine, purmorphamine and their corresponding vehicle controls at 24 hpf and 2 dpf, taken from the set of embryos analyzed in Figure 5. Treatment with 75 uM purmorphamine alone did not cause any abnormalities (last column; 24 hpf control MO plus 75 uM purmorphamine alone, n = 123; 2 dpf control MO plus 75 uM purmorphamine alone, n = 114, 3 independent biological repeats). (B) Suppression of Hh pathway with cyclopamine rescued the rod photoreceptor defect in *sox11* morphants at 3 dpf (right; number of embryos analyzed: *sox11* MO, n = 20; *sox11* MO + cyclopamine, n = 18; 3 independent repeats). (C) Retinal cell types were visualized by immunohistochemistry (ganglion, cones, amacrine, horizontal, and bipolar cells) or with a transgenic fluorescent reporter lines (Tg(gfap: GFP)mi2001) for Müller glia in *sox11* morphants (left) and *sox11* morphants treated with cyclopamine (right) at 3 dpf. The retinas of *sox11* morphants treated with cyclopamine were well laminated and displayed normal distributions of all cell types (n = 15 per group, 3 independent repeats). D, dorsal; V, ventral; A, anterior; P, posterior; MO, morpholino; hpf, hours post fertilization; L, lens.
(TIF)

Figure S5 Hh pathway gene expression changes in *sox11* morphants. (A) QPCR performed on mRNA from *sox11* morphant and control heads at 18 hpf reveal small increases in *gli2a* and *gli3* expression in *sox11* morphants compared to controls, but no significant change in *shha* expression. Relative transcript abundance was normalized to *atp5h* levels and is presented as the mean fold-change in expression relative to controls (B) At 24 hpf, *sox11* morphants demonstrated a large increase in *shha* expression, which correlated with the dose of *sox11* MO injected. Relative transcript abundance was normalized to *gapdh* levels and is presented as the mean fold-change in expression relative to controls (n = 60 embryos per group, 3 independent biological repeats) *p<0.01, Student's *t*-test. (C) Representative bright-field images of embryos injected with *sox11* MO alone (left side), *shha* MO alone (middle), or both *shha* and *sox11* MOs (right side), taken from the set of embryos analyzed in Figure 6E. (D) Co-knockdown of *shha* increased rod photoreceptor number in *sox11* morphants at 3 dpf (number of embryos analyzed: control MO, n = 11; *shha* MO, n = 10; *sox11* MO, n = 16, *sox11*+ *shha* MO, n = 15, 3 independent repeats) *p<0.05, Student's *t*-test. (E) QPCR performed on mRNA from heads of *sox11* morphants treated with vehicle (100% ethanol) or cyclopamine and compared to control morphants treated with vehicle revealed a significant reduction in *shha* expression in *sox11* morphants treated with cyclopamine at 24 hpf. Relative transcript abundance was normalized to *gapdh* levels and is presented as the mean fold-change in expression relative to controls (n = 40 embryos per group, 3 independent biological repeats) **p<0.0001, Student's *t*-test. (F) QPCR for *shha* was performed on mRNA from the 24 hpf heads of *sox11* morphants injected with half the normal dose and treated with DMSO, *sox11* morphants (half dose) treated with purmorphamine, and compared to control morphants treated with DMSO. An increase in *shha* expression was detected in *sox11* morphants (half dose) treated with purmorphamine compared to *sox11* morphants (half dose) treated with DMSO, however the increase did not reach the threshold for statistical significance. Relative transcript abundance was normalized to *gapdh* levels and is presented as the mean fold-change in expression relative to controls (n = 40 embryos per group, 3 independent biological repeats). (G) QPCR was performed on mRNA from the 24 hpf heads of zebrafish embryos injected with control (*td*-*tomato*) mRNA and embryos injected with zebrafish *sox11* mRNA. This analysis revealed a significant decrease in *shha* expression in embryos overexpressing zebrafish *sox11* mRNA compared to the controls. Relative transcript abundance was normalized to *18s* rRNA levels and is presented as the mean fold-change in expression relative to controls (n = 30 embryos per group, 3 independent biological repeats). **p<0.0001, Student's *t*-test. (H) QPCR performed on mRNA from heads of 24 hpf embryos injected with *shha* MO or control MO revealed no significant change in expression of either *sox11a* or *sox11b* in *shha* morphants compared to controls. Relative transcript abundance was normalized to *gapdh* levels and is presented as the mean fold-change in expression relative to controls (n = 45 embryos per group, 3 independent biological repeats). ns, p>0.05, Student's *t*-test. D, dorsal; V, ventral; A, anterior; P, posterior; MO, morpholino; hpf, hours post fertilization; dpf, days post fertilization; L, lens.
(TIF)

Figure S6 *Bmp7b* expression is reduced in *sox11* morphants. QPCR performed on mRNA from control and *sox11* morphants bodies (8–12 hpf) or heads (18–24 hpf) for *bmp7b* revealed a significant downregulation of *bmp7b* in *sox11* morphants at all time points except 18 hpf compared to controls. Relative transcript abundance was normalized to *atp5h* (18 hpf) and *gapdh* (8, 10, 12, and 24 hpf) levels and is presented as the mean fold-change in expression relative to controls (n = 50 embryos per group, 3 independent biological repeats). **p<0.01, *p<0.05, Student's *t* –test.
(TIF)

Figure S7 Sox4 compensates for the loss of Sox11. (A) *Sox4a* was diffusely expressed in the control retina at 36 hpf (left); however, *sox4a* expression was upregulated in the lens and retina of *sox11* morphants (right; n = 20 per group); scale bar = 50 µm. (B) QPCR performed on mRNA from the heads of 24 hpf zebrafish embryos injected with *sox11* MO or control MO reveal that *sox4a* expression is elevated in *sox11* morphants compared to controls. Relative transcript abundance was normalized to *gapdh* levels and is presented as the mean fold-change in expression relative to controls (n = 40 embryos per group, 3 independent biological repeats) *p<0.01, Student's *t*-test. (C) Co-injection of *sox4* mRNA rescued the lens and coloboma phenotypes of *sox11* morphants at 24 hpf and 2dpf. Number of embryos analyzed: 24 hpf control MO, n = 136; 2 dpf control MO, n = 124; 24 hpf *sox11* MO, n = 179; 2 dpf *sox11* MO, n = 161, 24 hpf *sox11* MO + *sox4* mRNA, n = 210, 2 dpf *sox11* MO + *sox4* mRNA, n = 184, 3 independent biological replicates. *p<0.001, Fishers exact test; MO, morpholino.
(TIF)

Figure S8 Association of *SOX11* locus with ocular abnormalities. (A) Amino acid sequence of human SOX11, with previously identified non-synonomous SNPs highlighted in green. The two variants identified in the MAC patients (positions indicated in red) are novel. (B) Scotopic ERG analysis of the proband's mother carrying the S315–354dup variant, demonstrating a reduction in the b-wave amplitude. (C) Western blot for SOX11 and β-actin in COS-7 cells transfected with SOX11 expression constructs. Densitometric analysis was performed with ImageJ software. (D) Representative brightfield images of *sox11* morphants co-injected with either WT, MI (G145C), or MII (S315–354dup) *SOX11* mRNA at 24 hpf and 2 dpf, taken from the set of embryos analyzed in Figure 8D. (E) Representative brightfield images of embryos overexpressing human WT, MI, or MII *SOX11* mRNA, taken from the set of embryos analyzed in Figure 8F. (F) Array CGH analysis of a proband with optic nerve agenesis and microphthalmia and her parents, confirming the presence of a de novo interstitial deletion at chromosome 2p25.2 (shaded gray). D, dorsal; V, ventral; A, anterior; P, posterior; MO, morpholino; hpf, hours post fertilization; dpf, days post fertilization; L, lens.
(TIF)

Table S1 Primer sequences used in this study.
(DOCX)
