# Supplementary figures and images for: Sox11 Is Required to Maintain Proper Levels of Hedgehog Signaling during Vertebrate Ocular Morphogenesis
Source: PLoS Genet. 2014 Jul 10;10(7):e1004491. doi: 10.1371/journal.pgen.1004491 (PMC4091786; doi:10.1371/journal.pgen.1004491)

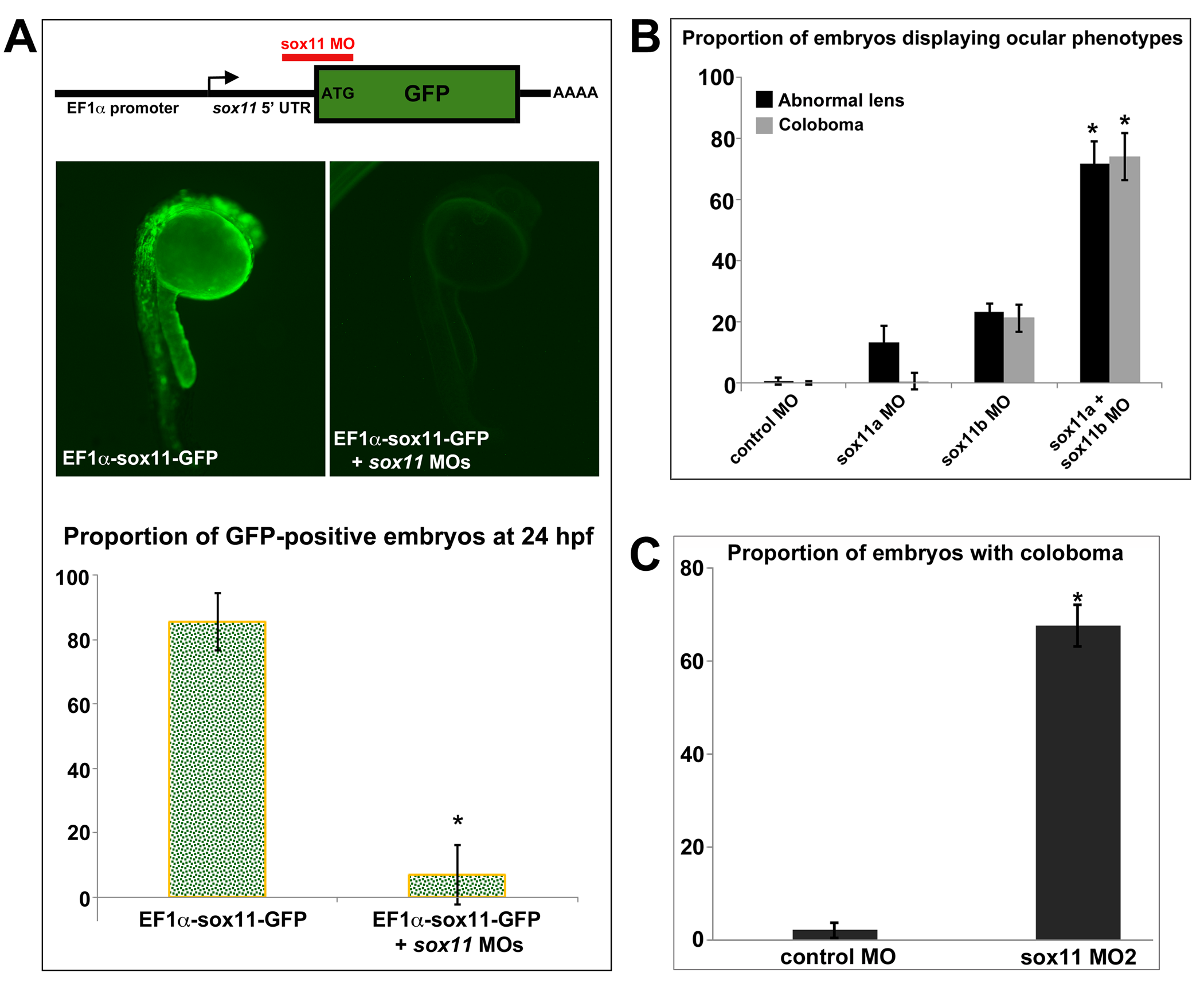

Supplement: Figure S1 — Efficiency and specificity of sox11 morpholinos. (A) Schematic representation of the pEF1α:GFP plasmid containing a portion of the sox11 5′ UTR placed upstream of the GFP reporter (top). The binding site for the sox11 morpholino is shown in red. Separate reporters were constructed for the sox11a and sox11b MOs. (Center) Lateral view (anterior at top) of 24 hpf embryos injected with EF1α- sox11a/b-GFP plasmids alone (left) or with both sox11 MOs. No GFP expression was detected in the embryo injected with sox11 MOs. (Bottom) Quantification of the proportion of GFP-positive embryos at 24 hpf. The sox11 MOs were highly effective at blocking GFP expression. Number of embryos analyzed: pEF1α-sox11-GFP plasmid alone, n = 169; pEF1α-sox11-GFP + sox11 MOs, n = 140, 3 independent repeats; *p = 0.004, Student's t-test. (B) Both sox11a and sox11b contribute to abnormal lens and coloboma phenotypes observed in sox11 morphants. The proportion of embryos displaying either phenotype was significantly higher when injected with sox11a and sox11b MOs simultaneously, compared to either MO alone. Number of embryos analyzed: 24 hpf control MO, n = 463; 2 dpf control MO, n = 441; 24 hpf sox11a MO, n = 229; 2 dpf sox11a MO, n = 214; 24 hpf sox11b MO, n = 341; 2 dpf sox11b MO, n = 316; 24 hpf sox11a + sox11b MO, n = 271; 2 dpf sox11a + sox11b MO, n = 262, 3 independent repeats. *p<0.001, Fisher's exact test. (C) A second non-overlapping sox11 MO (that targeted both sox11a and sox11b simultaneously) produced the same coloboma phenotype in similar proportion to the first set. Number of embryos analyzed: control MO, n = 186 embryos; sox11 MO, n = 194, 3 independent repeats; *p<0.001, Fisher's exact test. MO, morpholino; hpf, hours post fertilization; dpf, days post fertilization. (TIF) [file pgen.1004491.s001.tif]

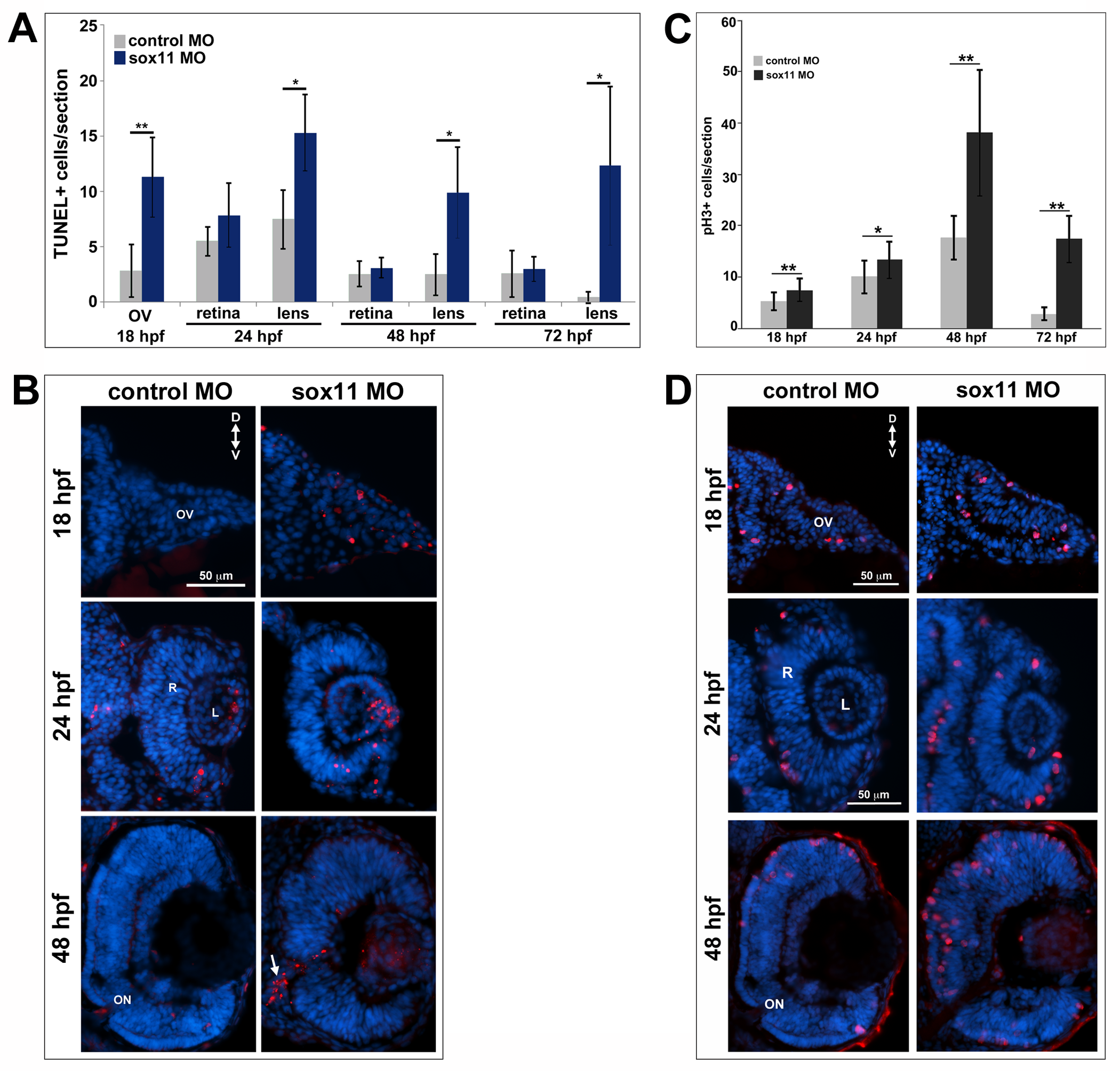

Supplement: Figure S2 — Cell proliferation and apoptosis in sox11 morphants. (A) Quantification of TUNEL+ cells in the optic vesicle, lens, and retina of control and sox11 and morphants from 18–72 hpf. Sox11 morphants had an elevated number of TUNEL+ cells in the optic vesicle at 18 hpf. Additionally, sox11 morphants consistently displayed more TUNEL+ cells in the anterior lens compared to controls from 24 -72 hpf. Number of embryos analyzed: 18 hpf control MO, n = 20; 18 hpf sox11 MO, n = 22; 24 hpf control MO, n = 15; 24 hpf sox11 MO, n = 19; 48 hpf control MO, n = 10; 48 hpf sox11 MO, n = 13; 72 hpf control MO, n = 12; 72 hpf sox11 MO, n = 12; average of 3 independent biological replicates. **p<0.00001, *p<0.01, Student's t-test. (B) Representative transverse sections of control (left column) and sox11 (right column) morphants at 18, 24, and 48 hpf, taken from the set of individuals analyzed in (A). At 48 hpf, TUNEL+ cells were detected within the colobomatous tissue and the region of the optic stalk in sox11 morphants (arrow, bottom right). (C) Sox11 morphant retinas had more PH3+ cells than controls from 18–72 hpf. Number of embryos analyzed: 18 hpf control MO, n = 12; 18 hpf sox11 MO, n = 15; 24 hpf control MO, n = 20; 24 hpf sox11 MO, n = 19; 48 hpf control MO, n = 10; 48 hpf sox11 MO, n = 12; 72 hpf control MO, n = 14; 72 hpf sox11 MO, n = 12; average of 3 independent biological replicates. **p<0.001, *p<0.01, Student's t-test. (D) Representative transverse sections of control (left column) and sox11 (right column) morphants at 18, 24, and 48 hpf, taken from the set of individuals analyzed in (C). D, dorsal; V, ventral; MO, morpholino; hpf, hours post fertilization; ON; optic nerve; OV, optic vesicle; R, retina; L, lens. (TIF) [file pgen.1004491.s002.tif]

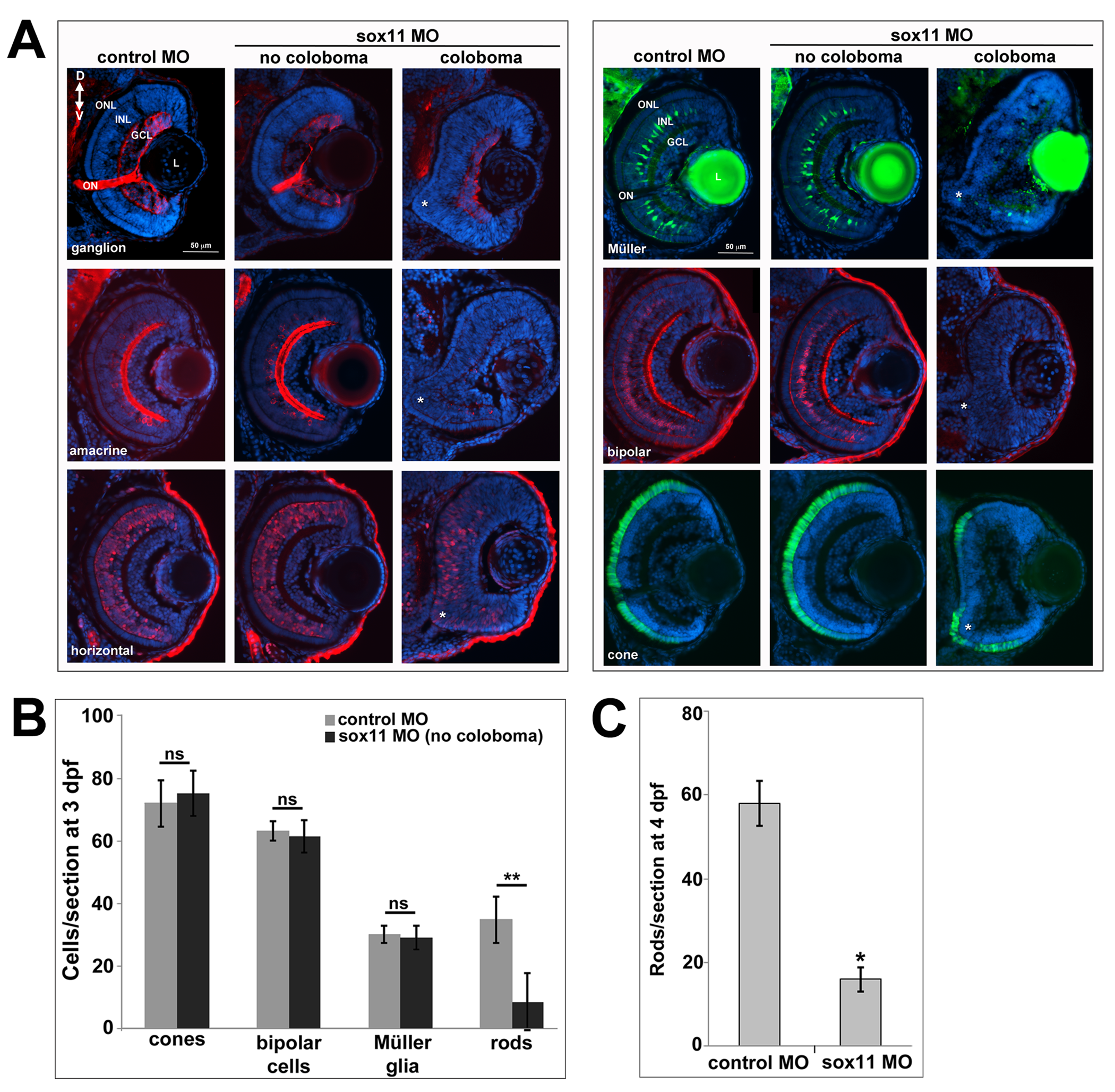

Supplement: Figure S3 — Retinal neurogenesis in sox11 morphants. (A) Retinal cell types were visualized by immunohistochemistry (ganglion, amacrine, horizontal, and bipolar cells) or with fluorescent reporter transgenic lines (Tg(gfap:GFP)mi2001 for Müller glia and Tg(3.2TαC-EGFP) for cones) in controls (left) and sox11 morphants (center, right) at 3 dpf. In sox11 morphants without coloboma (center), the retinas are well laminated and had normal numbers of ganglion, amacrine, horizontal, and bipolar cells, cone photoreceptors, and Müller glia. However, sox11 morphants with coloboma (asterisk; right) had poorly laminated retinas and reduced numbers of differentiated retinal cell types, indicating delayed retinal development. (B) Quantification of numbers of late-born retinal cell types in control and sox11 morphants without coloboma. Only rod photoreceptors displayed a significant reduction. Number of embryos analyzed: control MO, n = 19; sox11 MO without coloboma, n = 25, 3 independent repeats.**p<0.00001; ns = p>0.05, Student's t-test.(C) At 4 dpf, sox11 morphants have more mature rod photoreceptors than at 3 dpf but the number remains significantly less than controls (*p<0.001, Student's t-test); MO, morpholino; dpf; days post fertilization; L, lens; GCL, ganglion cell layer; INL, inner nuclear layer; ONL, outer nuclear layer; ON, optic nerve. (TIF) [file pgen.1004491.s003.tif]

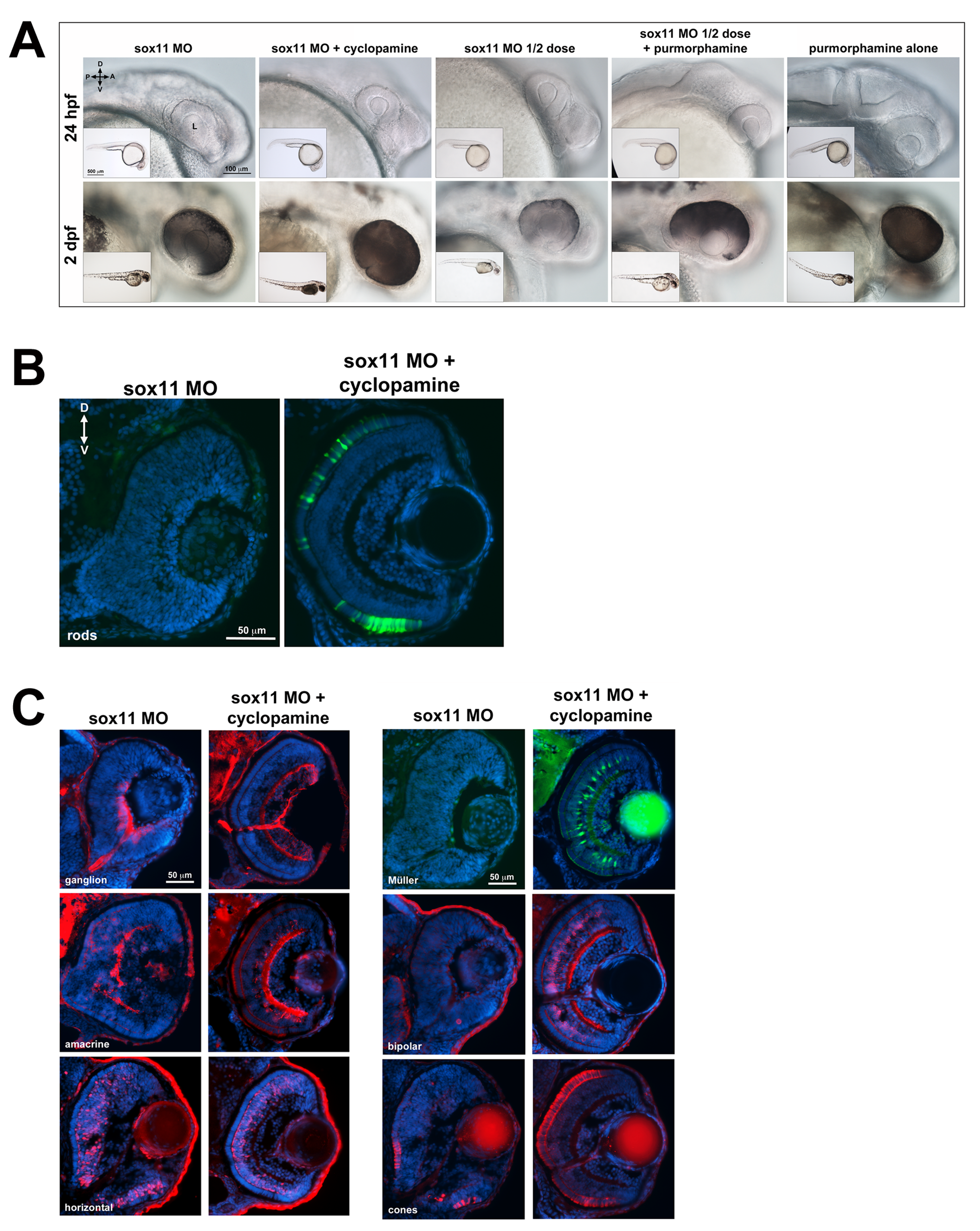

Supplement: Figure S4 — Elevated Hh signaling contributes to the abnormal ocular phenotypes displayed by sox11 morphants. (A) Representative brightfield images of sox11 morphants treated with cyclopamine, purmorphamine and their corresponding vehicle controls at 24 hpf and 2 dpf, taken from the set of embryos analyzed in Figure 5. Treatment with 75 uM purmorphamine alone did not cause any abnormalities (last column; 24 hpf control MO plus 75 uM purmorphamine alone, n = 123; 2 dpf control MO plus 75 uM purmorphamine alone, n = 114, 3 independent biological repeats). (B) Suppression of Hh pathway with cyclopamine rescued the rod photoreceptor defect in sox11 morphants at 3 dpf (right; number of embryos analyzed: sox11 MO, n = 20; sox11 MO + cyclopamine, n = 18; 3 independent repeats). (C) Retinal cell types were visualized by immunohistochemistry (ganglion, cones, amacrine, horizontal, and bipolar cells) or with a transgenic fluorescent reporter lines (Tg(gfap: GFP)mi2001) for Müller glia in sox11 morphants (left) and sox11 morphants treated with cyclopamine (right) at 3 dpf. The retinas of sox11 morphants treated with cyclopamine were well laminated and displayed normal distributions of all cell types (n = 15 per group, 3 independent repeats). D, dorsal; V, ventral; A, anterior; P, posterior; MO, morpholino; hpf, hours post fertilization; L, lens. (TIF) [file pgen.1004491.s004.tif]

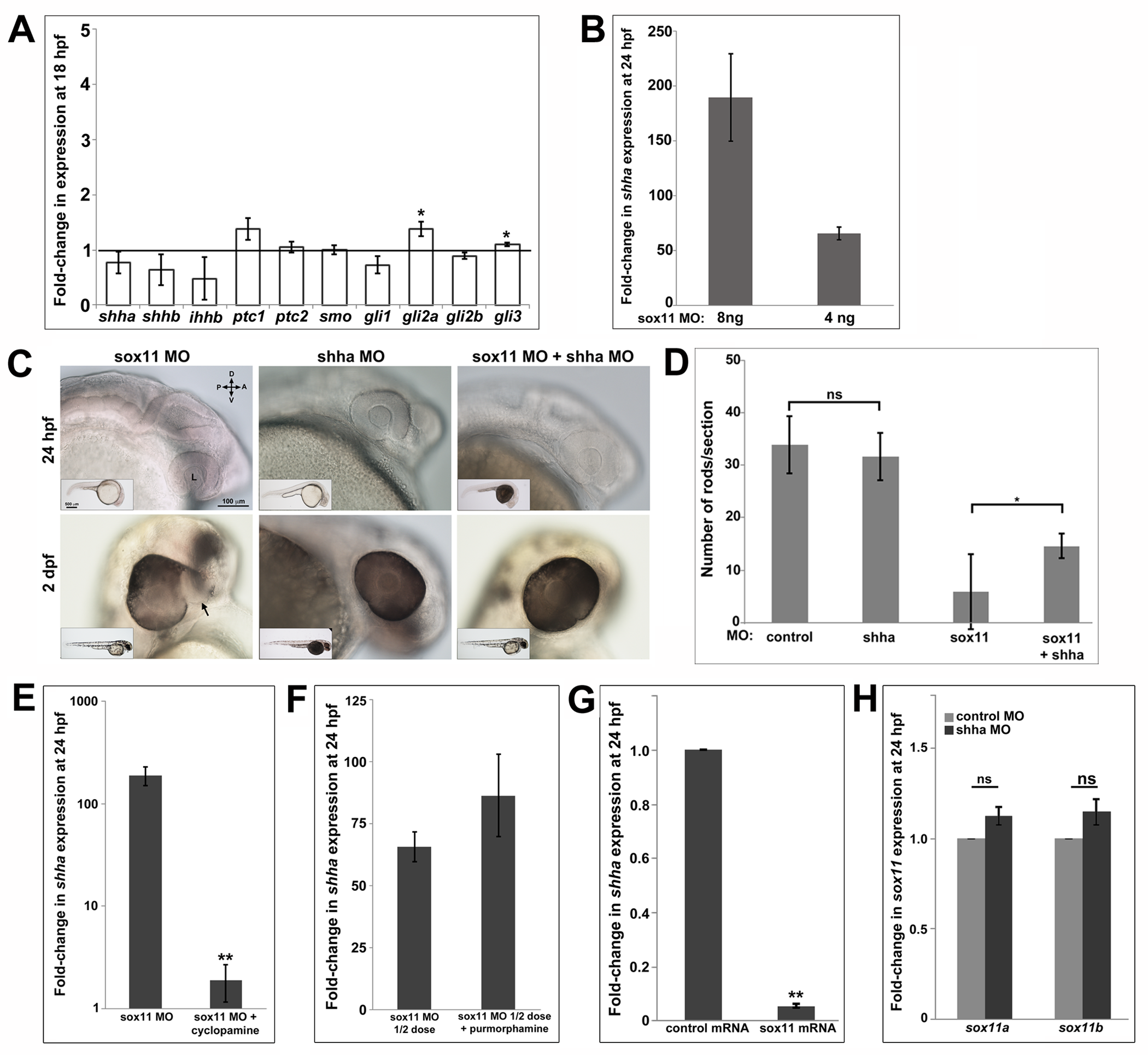

Supplement: Figure S5 — Hh pathway gene expression changes in sox11 morphants. (A) QPCR performed on mRNA from sox11 morphant and control heads at 18 hpf reveal small increases in gli2a and gli3 expression in sox11 morphants compared to controls, but no significant change in shha expression. Relative transcript abundance was normalized to atp5h levels and is presented as the mean fold-change in expression relative to controls (B) At 24 hpf, sox11 morphants demonstrated a large increase in shha expression, which correlated with the dose of sox11 MO injected. Relative transcript abundance was normalized to gapdh levels and is presented as the mean fold-change in expression relative to controls (n = 60 embryos per group, 3 independent biological repeats) *p<0.01, Student's t-test. (C) Representative bright-field images of embryos injected with sox11 MO alone (left side), shha MO alone (middle), or both shha and sox11 MOs (right side), taken from the set of embryos analyzed in Figure 6E. (D) Co-knockdown of shha increased rod photoreceptor number in sox11 morphants at 3 dpf (number of embryos analyzed: control MO, n = 11; shha MO, n = 10; sox11 MO, n = 16, sox11+ shha MO, n = 15, 3 independent repeats) *p<0.05, Student's t-test. (E) QPCR performed on mRNA from heads of sox11 morphants treated with vehicle (100% ethanol) or cyclopamine and compared to control morphants treated with vehicle revealed a significant reduction in shha expression in sox11 morphants treated with cyclopamine at 24 hpf. Relative transcript abundance was normalized to gapdh levels and is presented as the mean fold-change in expression relative to controls (n = 40 embryos per group, 3 independent biological repeats) **p<0.0001, Student's t-test. (F) QPCR for shha was performed on mRNA from the 24 hpf heads of sox11 morphants injected with half the normal dose and treated with DMSO, sox11 morphants (half dose) treated with purmorphamine, and compared to control morphants treated with DMSO. An increase in shha expression wa [file pgen.1004491.s005.tif]

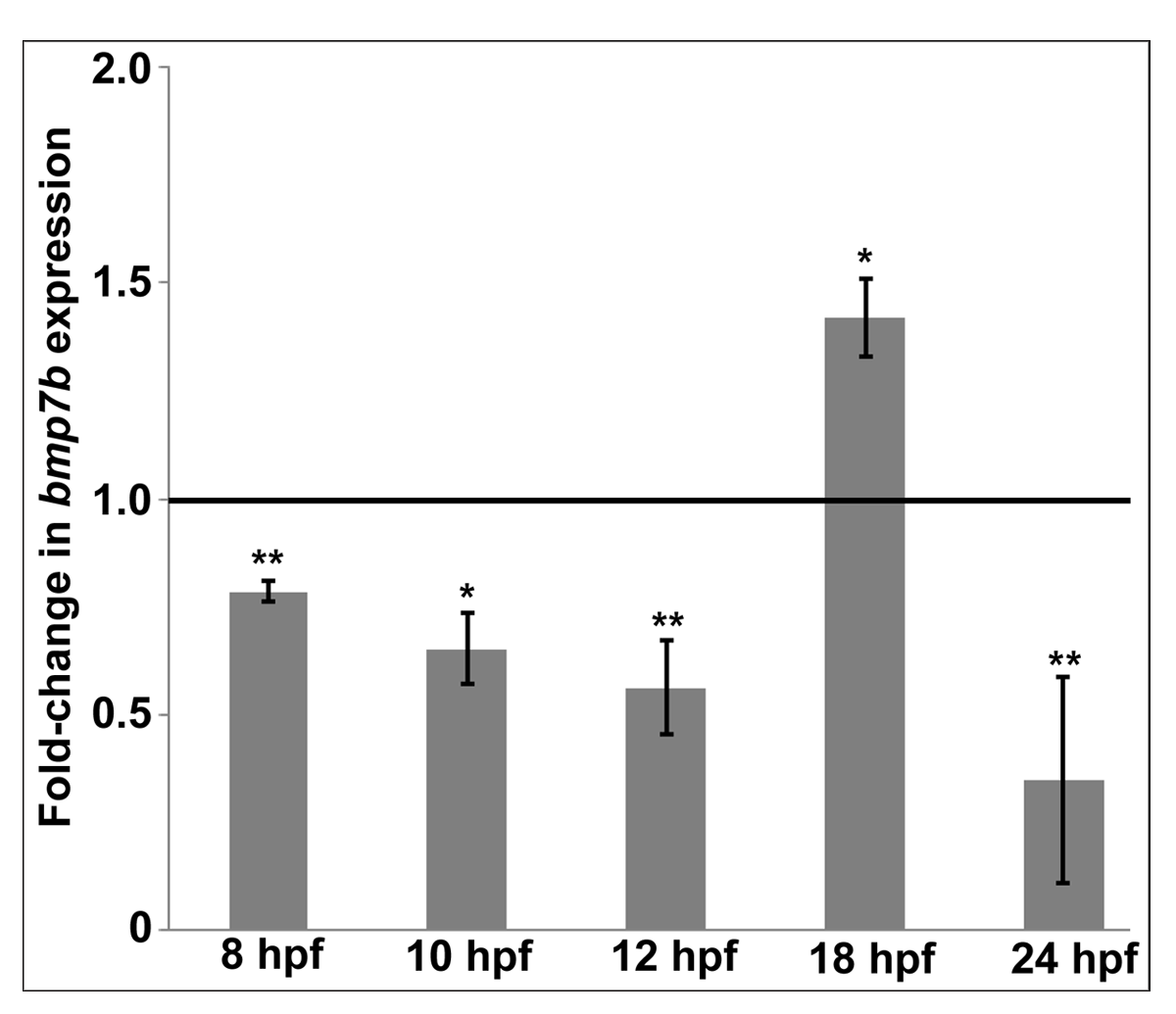

Supplement: Figure S6 — Bmp7b expression is reduced in sox11 morphants. QPCR performed on mRNA from control and sox11 morphants bodies (8–12 hpf) or heads (18–24 hpf) for bmp7b revealed a significant downregulation of bmp7b in sox11 morphants at all time points except 18 hpf compared to controls. Relative transcript abundance was normalized to atp5h (18 hpf) and gapdh (8, 10, 12, and 24 hpf) levels and is presented as the mean fold-change in expression relative to controls (n = 50 embryos per group, 3 independent biological repeats). **p<0.01, *p<0.05, Student's t –test. (TIF) [file pgen.1004491.s006.tif]

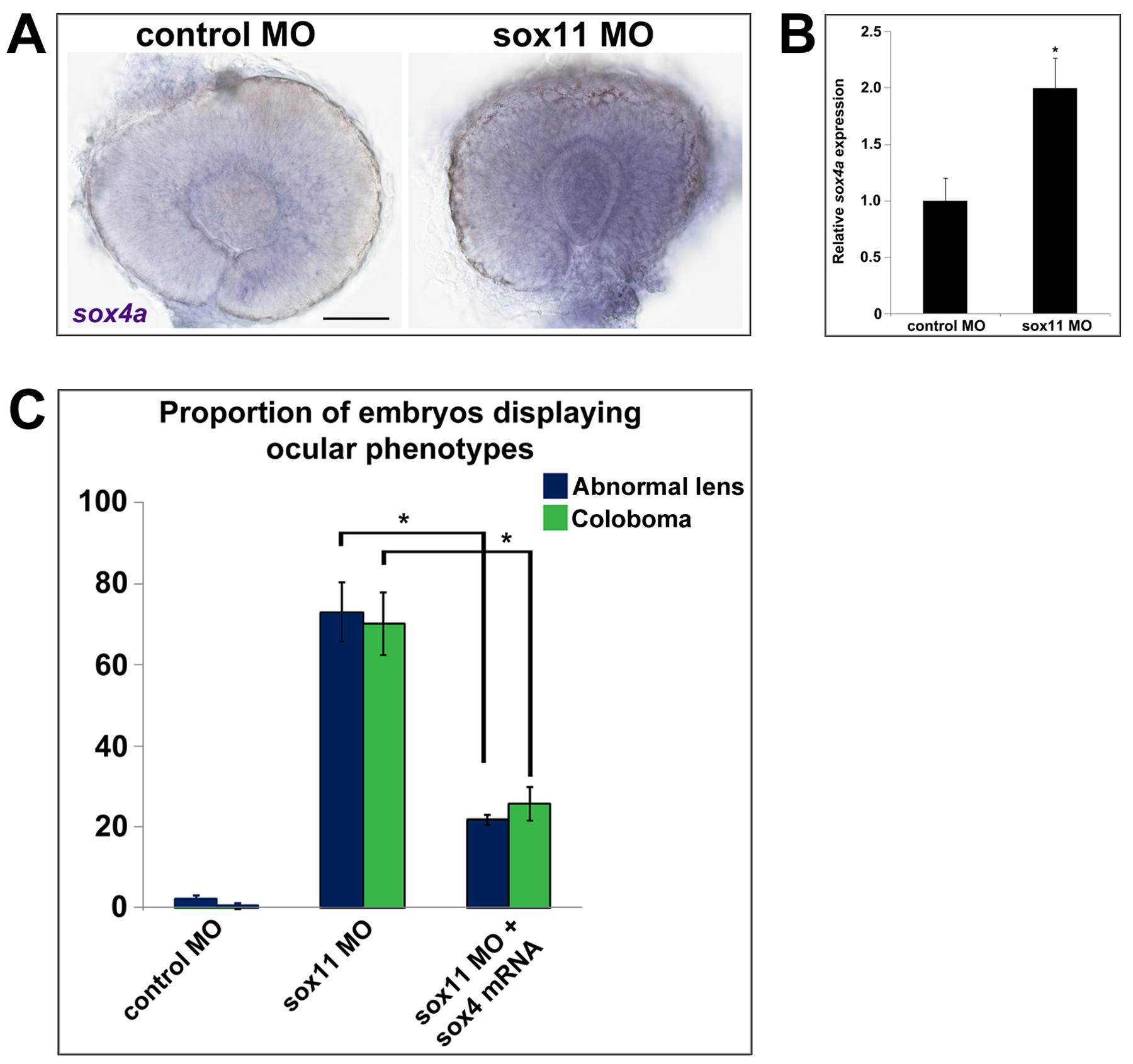

Supplement: Figure S7 — Sox4 compensates for the loss of Sox11. (A) Sox4a was diffusely expressed in the control retina at 36 hpf (left); however, sox4a expression was upregulated in the lens and retina of sox11 morphants (right; n = 20 per group); scale bar = 50 µm. (B) QPCR performed on mRNA from the heads of 24 hpf zebrafish embryos injected with sox11 MO or control MO reveal that sox4a expression is elevated in sox11 morphants compared to controls. Relative transcript abundance was normalized to gapdh levels and is presented as the mean fold-change in expression relative to controls (n = 40 embryos per group, 3 independent biological repeats) *p<0.01, Student's t-test. (C) Co-injection of sox4 mRNA rescued the lens and coloboma phenotypes of sox11 morphants at 24 hpf and 2dpf. Number of embryos analyzed: 24 hpf control MO, n = 136; 2 dpf control MO, n = 124; 24 hpf sox11 MO, n = 179; 2 dpf sox11 MO, n = 161, 24 hpf sox11 MO + sox4 mRNA, n = 210, 2 dpf sox11 MO + sox4 mRNA, n = 184, 3 independent biological replicates. *p<0.001, Fishers exact test; MO, morpholino. (TIF) [file pgen.1004491.s007.tif]

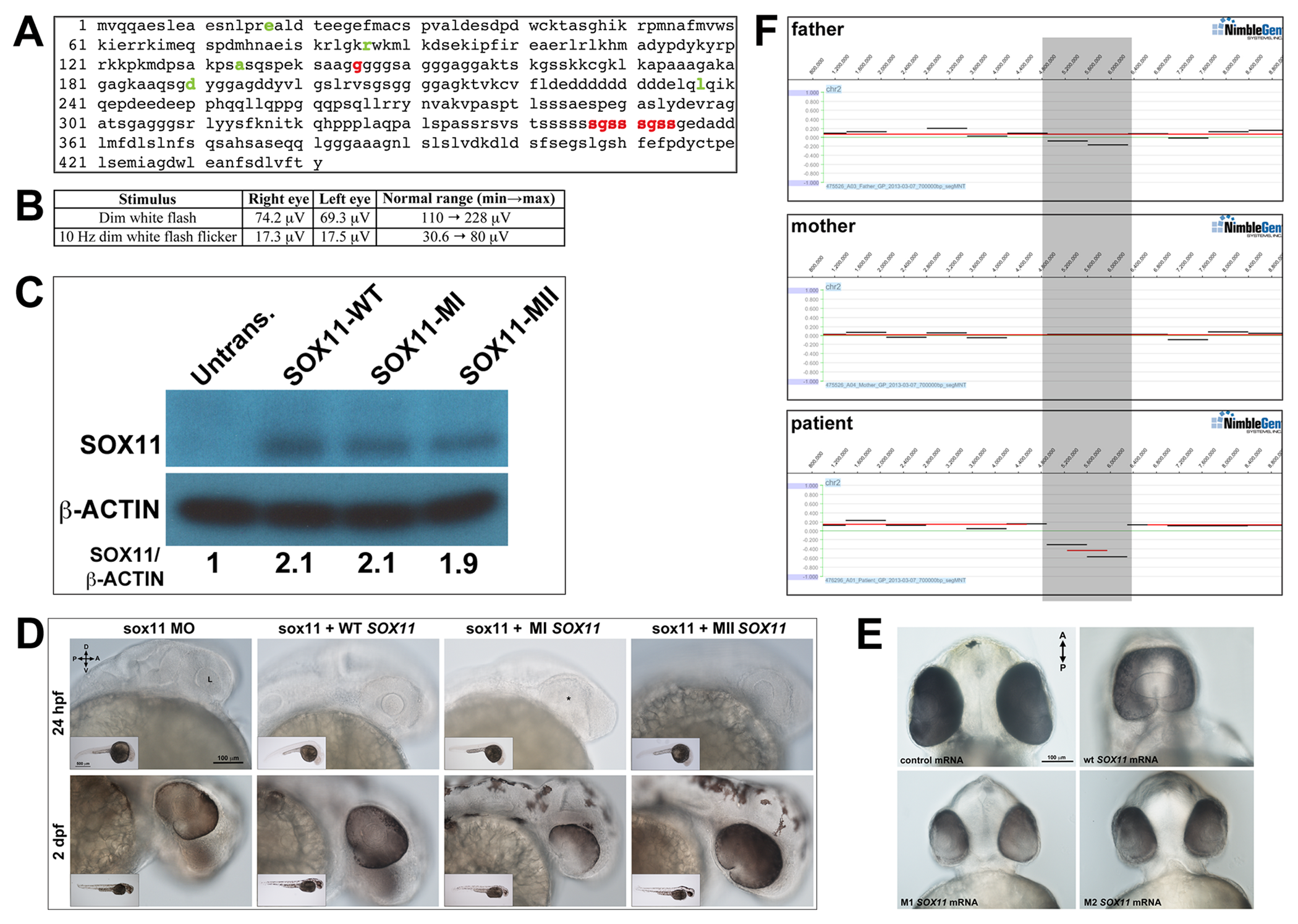

Supplement: Figure S8 — Association of SOX11 locus with ocular abnormalities. (A) Amino acid sequence of human SOX11, with previously identified non-synonomous SNPs highlighted in green. The two variants identified in the MAC patients (positions indicated in red) are novel. (B) Scotopic ERG analysis of the proband's mother carrying the S315–354dup variant, demonstrating a reduction in the b-wave amplitude. (C) Western blot for SOX11 and β-actin in COS-7 cells transfected with SOX11 expression constructs. Densitometric analysis was performed with ImageJ software. (D) Representative brightfield images of sox11 morphants co-injected with either WT, MI (G145C), or MII (S315–354dup) SOX11 mRNA at 24 hpf and 2 dpf, taken from the set of embryos analyzed in Figure 8D. (E) Representative brightfield images of embryos overexpressing human WT, MI, or MII SOX11 mRNA, taken from the set of embryos analyzed in Figure 8F. (F) Array CGH analysis of a proband with optic nerve agenesis and microphthalmia and her parents, confirming the presence of a de novo interstitial deletion at chromosome 2p25.2 (shaded gray). D, dorsal; V, ventral; A, anterior; P, posterior; MO, morpholino; hpf, hours post fertilization; dpf, days post fertilization; L, lens. (TIF) [file pgen.1004491.s008.tif]
