# Supplementary material for: Sox11 Is Required to Maintain Proper Levels of Hedgehog Signaling during Vertebrate Ocular Morphogenesis
Source: PLoS Genet. 2014 Jul 10;10(7):e1004491. doi: 10.1371/journal.pgen.1004491 (PMC4091786; doi:10.1371/journal.pgen.1004491)
Supplement: Table S1 — Primer sequences used in this study. (DOCX) [file pgen.1004491.s009.docx]

| **Gene** | **Forward primer (5'→3')** | **Reverse primer (5'→3')** | **Use** |
| --- | --- | --- | --- |
| *sox11a* [21] | CCCACTTTGAATTTCCAGACTATTG | CTTTGCTATACTTTTGACGTGTCTCC | probe |
| *sox11b* | GCTGGCACATTCTCCAATTT | TTGCTACAAAACGCAGATGG | probe |
| *sox11a* | ATGGTGCAGCAAACGGAC | CCTCTCTCCCGTGTGTGTT | overexpression |
| *sox11b* | ACATGGTGCAGCAGACGGA | ATGCCTCCTTGGTCATGGA | overexpression |
| *sox11a* | AACTGCAAGTAATTCAGATG | CCTGGTTACTGTCCATTGT | qPCR |
| *sox11b* | CAGGTTATTTTTTGACTCTGCCT | GTCAATGTTTGGACCATGTT | qPCR |
| *sox11a* 5’UTR | GGTACCGGCACGAGGAGTCGCAGAG | CCCGGGTCCGTTTCGCTGTTGTCC | cloning |
| *sox11b* 5’ UTR | GGTACCCAAGAGGGAAAAGTGTGTT | CCCGGGATCATTTCGCTCTCCTCGGT | cloning |
| *shha* | ACACGCGTGAACCGAGGAG | GTGTCATGAGCCTGTCCGC | probe |
| *SOX11* I | ACCTCCGCACGAGACCCAG | GCATTTCTTGCTGGAGCCCT | sequencing |
| *SOX11* II | GCCCAGAGAAGAGCGCGG | GAGGTGGACACCGAGCGC | sequencing |
| *SOX11* III | AACATCACCAAGCAGCAGCACCC | GAACCCAGCTCTGCACCCT | sequencing |
| *SOX11* RFLP I | GGCCCTGGACGAGAGCGAC | ACGTAGTCGTCGCCCGCGC | PCR |
| *SOX11* RFLP II | GTCGCAGCTGCTGAGACGCTACAA | GCTGTGCGCGCTTTGAGAGA | PCR |
| *SOX11* full length | AAGCTTACCTCCGCACGAGACCCAG | CTCGAGGAACCCAGCTCTGCACCCT | cloning |
| *shha* | CCAGGTTCGGCTCTGGTCTC | AGGTTTCCCGCGCTGTCTG | qPCR |
| *shhb* | ACACATGCGAGAGCCTCAA | CAAGAGGTGAGCACATCGTT | qPCR |
| *ihhb* | TCAAGTGGGTCAGTGTTTG | CAAGAGGTGAGCACATCGTT | qPCR |
| *ptc1* | GTGCCGGTAAATTCCTCTCA | CGCATAGGCAAGCATTAGCA | qPCR |
| *ptc2* | GAAACTATGGGTGGAAGCT | CACTTGAACTTTGCTAGCTG | qPCR |
| *smo* | AGTTTGGCCCCAGTGCAGT | GGGAAGGCTCCAGGTAGCAT | qPCR |
| *gli1* | AGCAGTGCGGATCTGATGC | TAGCTTCGGTCTCCACCTGG | qPCR |
| *gi2a* | TCCACACATGGAGCATTAC | TACTCTGAAGGGTTTGCTC | qPCR |
| *gli2b* | CCAGAAGGTGCAGTGACAC | CGTTACTCTCAGACGTGCTG | qPCR |
| *gli3* | GGAACACTGTTTGCCATGGA | TGATGGTACTGGCGAAGGTT | qPCR |
| *sox4a* | TCTTAGCGCTTTCAACGCG | TGCTCCACCATAGCCAGGT | qPCR |
| *gapdh* | TGCTGGTATTGCTCTCAAC | GAGAATGGTCGCGTATCAA | qPCR |
| *atp5h* | TGCCATCTCAGCAAAACTTG | CACAGGCTCAGGAACAGTCA | qPCR |
| *18s ribosomal* RNA[78] | TCGCTAGTTGGCATCGTTTATG | CGGAGGTTCGAAGACGATCA | qPCR |
| *bmp7b* | GTGGGGATACTGTGTCCTGGC | ACAGGATCTCCCGCTGCATC | qPCR |

**Table S1. Primer sequences used in this study**
